# Predicting episodic memory formation for movie events

**DOI:** 10.1038/srep30175

**Published:** 2016-09-30

**Authors:** Hanlin Tang, Jed Singer, Matias J. Ison, Gnel Pivazyan, Melissa Romaine, Rosa Frias, Elizabeth Meller, Adrianna Boulin, James Carroll, Victoria Perron, Sarah Dowcett, Marlise Arellano, Gabriel Kreiman

**Affiliations:** 1 Children’s Hospital, Harvard Medical School, Boston, MA 02115, USA; 2 Program in Biophysics, Harvard University, Cambridge, MA 02138, USA; 3 School of Psychology, University of Nottingham, University Park, Nottingham, NG7 2RD, United Kingdom.; 4 University of California, Berkeley, CA, USA.; 5 Emmanuel College, Boston, MA 02115, USA

## Abstract

Episodic memories are long lasting and full of detail, yet imperfect and malleable. We quantitatively evaluated recollection of short audiovisual segments from movies as a proxy to real-life memory formation in 161 subjects at 15 minutes up to a year after encoding. Memories were reproducible within and across individuals, showed the typical decay with time elapsed between encoding and testing, were fallible yet accurate, and were insensitive to low-level stimulus manipulations but sensitive to high-level stimulus properties. Remarkably, memorability was also high for single movie frames, even one year post-encoding. To evaluate what determines the efficacy of long-term memory formation, we developed an extensive set of content annotations that included actions, emotional valence, visual cues and auditory cues. These annotations enabled us to document the content properties that showed a stronger correlation with recognition memory and to build a machine-learning computational model that accounted for episodic memory formation in single events for group averages and individual subjects with an accuracy of up to 80%. These results provide initial steps towards the development of a quantitative computational theory capable of explaining the subjective filtering steps that lead to how humans learn and consolidate memories.

Episodic memories constitute the essential fabric of our recollections. Our brains are continuously bombarded with external information but only a small fraction of these inputs is crystallized into episodic memories. There has been extensive work demonstrating that memories do not constitute a mere copy of input signals. Instead, the brain selects and interprets incoming inputs to actively construct a narrative that forms the basis of episodic memories (e.g. refs 1, 2, 3, 4, 5, 6).

To study the formation of episodic memories under natural conditions, it is necessary to systematically define each episodic event and a mechanism to evaluate those memories. The extent of memory recall versus failure depends on multiple factors including some which are intrinsic to the subjects themselves (e.g. age, domain knowledge), what contents are evaluated (e.g. single items versus episodic events, meaning and context, degree of similarity between targets and foils), when memory is probed (particularly the time between encoding and testing) and how recollection is evaluated (e.g. objective versus subjective metrics)^2^,^7^,^8^.

Most studies in the field have focused on recollection of words, faces, objects or scenes (e.g. refs 9, 10, 11, 12, 13, 14, 15), without considering the temporal and spatial context which is critical to real life memories. To understand memory formation under natural conditions, it is critical to incorporate the temporal and spatial contexts that lead to episodic events. One approach in this direction has focused on recollection of specific information within narratives^4^,^5^,^16^,^17^. While several heroic efforts have examined recollection for real-life memories (e.g. refs 4,10,18, 19, 20), it is often difficult to systematically study real-life events due to the challenges involved in establishing ground truth, reproducibility, appropriate controls, amount of practice or exposure and other variables.

An interesting alternative to examining memories for real-life events involves using movies as stimuli^21^,^22^,^23^. Movies contain several important aspects of episodic information that are difficult to deduce from single item studies including temporal sequences, spatial and temporal context, affective components and an underlying narrative. Subjects can form vivid and detailed memories for movie events as assessed by cued recall, recognition and metamemory confidence estimates^22^,^23^.

There have been significant advances in our theoretical understanding of memory, including the brain structures that play a central role in memory formation (e.g. refs 24, 25, 26, 27, 28). Yet, we still lack computational models implemented in functional algorithms that can explain what dynamic events will be remembered and make quantitative predictions about how subjects learn and form new memories. In order to quantitatively examine the relationship between event contents and the filtering events that lead to memory formation under dynamic real world (or close to real world) scenarios, here we systematically investigated the robustness of long-term episodic memory formation for movie events. We quantitatively characterized the content variables that dictate the formation of episodic memories at retention times of up to one year by combining extensive psychophysics measurements and a large set of stimulus annotations. Next, we used a machine learning approach to demonstrate that a computational algorithm based exclusively on visual, auditory and emotional content can predict what individual subjects or groups of subjects do and do not remember from a movie. The computational methodology discussed here was recently presented during the 50^th^ Annual Conference on Information Sciences and Systems^29^.

## Results

**Figure 1:**
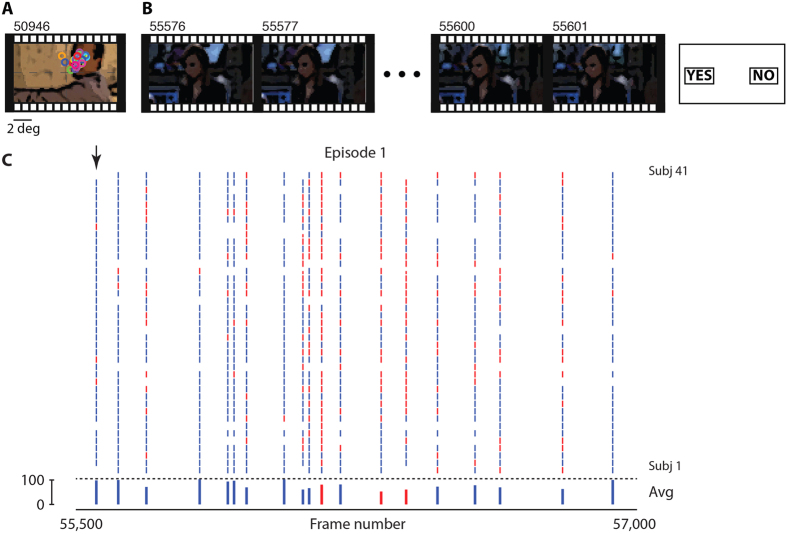
Experimental design and performance consistency. (**A**) Single frame (frame 50946) from the movie showing eye fixations from 25 subjects (each colored circle denotes a separate subject). Note that owing to copyright problems, all original images have been replaced in this and all subsequent figures by very similar artistic renderings. (**B**) During each recognition memory trial, subjects were presented with a single shot (here from frame 55576 to frame 55601, duration = 0.833 seconds). Subjects indicated whether or not they had seen the events in the shot during the movie. (**C**) Raster plot showing the performance of each of the 41 subjects (one subject per row) for multiple shots from frame 55,500 to frame 56,800. Each vertical mark indicates the subject’s response (blue = correct, red = incorrect). Bottom: for each shot, if most subjects were correct, the height of the blue line indicates the percentage of subjects that were correct; if most subjects were incorrect, the height of the red line indicates the percentage of subjects that were incorrect (see Fig. S2 for a raster over the whole experiment).

We sought to systematically and quantitatively evaluate the internal and subjective filtering events that dictate long-term memorability of episodic events during a movie. In the main experiment, forty-one subjects watched a 42-minute movie (a TV series named “24”, Season 6, Episode 1) while we monitored their eye movements (Figs 1A and S1). Memory for specific episodic content was evaluated in 6 sessions, conducted 15 minutes to 365 days after subjects watched the movie. Memorability was evaluated by presenting brief movie shots lasting between 1 and 90 frames (Methods, Fig. 1B). These movie shots were defined as the sequence of frames separated by cuts denoting large changes between consecutive frames (Fig. S1A). We extensively sampled recognition memory across the movie using randomly interleaved query shots.

During the recognition memory tests, shots from the target movie were intermixed with an equal proportion of foil shots from the next episode in the same TV series (Episode 2), which the subjects had not watched. The events during these two episodes are purported to take place during two consecutive hours of the day and therefore characters are typically wearing the same clothes, the locations and basic settings are similar, the filming style is the same, etc. Furthermore, the control shots from Episode 2 were matched to those in Episode 1 in terms of duration and visual content (Fig. S1B, Methods). Subjects performed an old/new task indicating whether they had seen the events in each shot during the movie presentation or not.

We summarize performance during the recognition memory tests by reporting the percentage of trials when subjects were correct (chance level = 50%). The overall percentage of correct trials combines the probability of hits (the probability of reporting a correct answer when the target was shown) and the probability of false alarms (the probability of reporting an incorrect answer when the foil was shown).

**Figure 2:**
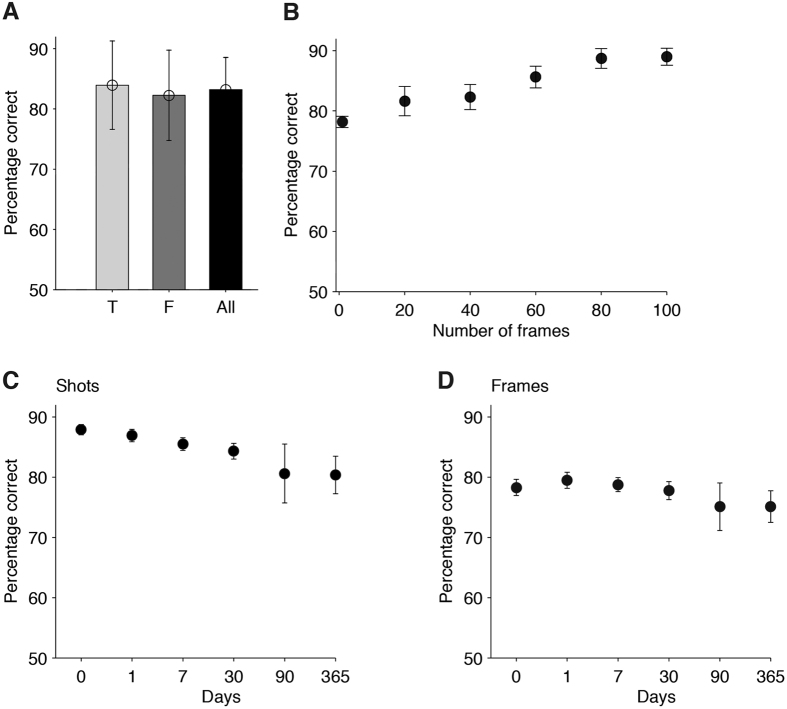
Performance increased with the number of frames and decreased with elapsed time after encoding. (**A**) Overall performance (mean ± SD, n = 41 subjects). There was no significant difference in overall performance between target trials (T) or foil trials (F) (non-parametric permutation test, *p* = 0.17). (**B**) Performance increased with the number of frames in the shot (*r* = 0.97, *p* < 10^−10^). Bin size = 30 frames; results are shown in the center of each bin. (**C**) Performance for shots decreased with elapsed time after encoding (*r* = −0.96, *p* = 0.001). Note that the scale on the x-axis is not linear in time (test points are shown at equidistant intervals along the x-axis). (**D**) Same as (**C**), showing performance for individual frames (*r* = −0.86, *p* = 0.018).

Overall, subjects were correct in 85.6 ± 5.3% of the trials (mean ± SD). This level of performance was statistically above chance levels (50%) and below ceiling levels (100%) (*p* < 10^−14^, permutation test), providing an ample range to investigate which variables contribute to recognition memory. All subjects performed well above chance and below ceiling (Fig. S2). There was no significant difference in performance between target and foil trials (86.6 ± 6.8% versus 84.4 ± 7.8% respectively, *p* = 0.17, permutation test, Fig. 2A; in subsequent analyses and unless otherwise stated, data from target and foil trials were pooled). While overall recognition memory for shots lasting several tens of frames (30 frames/sec) could be expected based on everyday subjective experience and previous studies (e.g. refs 22,23), subjects also performed well above chance levels in trials containing only one frame (referred to as single frames, 78.2 ± 6.0%). A recent study has also demonstrated the ability to correctly discriminate old versus novel frames in movie streams using shorter intervals between encoding and testing^21^. The high performance in correctly recognizing single frames is reminiscent of work demonstrating a significant capacity to remember object details in single item studies^11^,^12^. The results reported here extend previous studies by demonstrating high memorability for shots and individual frames in movie events where targets and foils are similar across two episodes in a movie as well as high memorability in situations that are close to real life where the stimuli are embedded in complex spatiotemporal context dictated by the movie, as opposed to studies of single items.

Responses in individual trials were consistent (i.e., reproducible across repetitions of the same query), both *within* and *between* subjects. Subjects responded self-consistently in repeat trials of the same shot (Fig. S4A–D). Above chance levels of self-consistency would be expected merely from above chance overall performance (in the extreme case, a subject who was 100% correct would always be self-consistent). Yet, subjects were more self-consistent than expected under the null hypothesis of independence after considering the overall performance (Fig. S4A–D, Methods).

There was also strong consistency *between* subjects (Figs 1C, S2, S3 and S4E–K). Examples of consistently correct and consistently incorrect answers in response to specific shots are shown in Fig. S3. Between-subject consistency was evident when comparing each subject to the mode response of all other subjects (Fig. S4E–H) and also when comparing subjects in a pairwise fashion (Fig. S4I–K). There was stronger between-subject consistency than expected under the null hypothesis of independence after considering the overall performance of each subject (Fig. S4E–K).

Performance increased with the duration of each shot, reaching approximately 90% for shots lasting ~3 seconds (Fig. 2B, p < 10^−10^, permutation test). Performance showed a significant decrease with the amount of time elapsed between encoding (movie watching) and the recognition memory test for both shots (Fig. 2C, p < 0.001) and individual frames (Fig. 2D, p = 0.018), consistent with a large body of previous studies on the retention function based on single items, narratives or autobiographical information (e.g. ref. 10). Remarkably, performance was above chance for single frames even when evaluated one year after encoding (75.1 ± 4.2%, p < 0.001).

To evaluate the degree of generalization in the results, we repeated the same experiment, in a different set of 22 subjects, but showing Episode 2 of the same TV series during encoding and using foils from Episode 1 (Variant 1, Table 1, Methods). None of the conclusions were altered in this experiment variation (Table S1); the overall performance was 82.5 ± 7.0% (cf. 85.6 ± 5.3% in the Main experiment).

In the Main experiment as well as in Variant 1, the same subjects were repeatedly tested in multiple sessions spanning multiple days to months. Even though no feedback was provided on their performance, and even though the shots were different across test sessions, this led to repeated exposure to the events during the movie. We performed a separate experiment variation in a different set of 37 subjects that were only tested during a single session (Variant 2, Table 1). Performance in this experiment variation was lower, 79.2 ± 5.9%, (cf. 85.6 ± 5.3% in the Main experiment), suggesting that there was a small but significant effect of unsupervised performance improvement due to repeated exposure. Other than these quantitative differences, all the qualitative conclusions were similar in experiment Variant 2.

**Figure 3:**
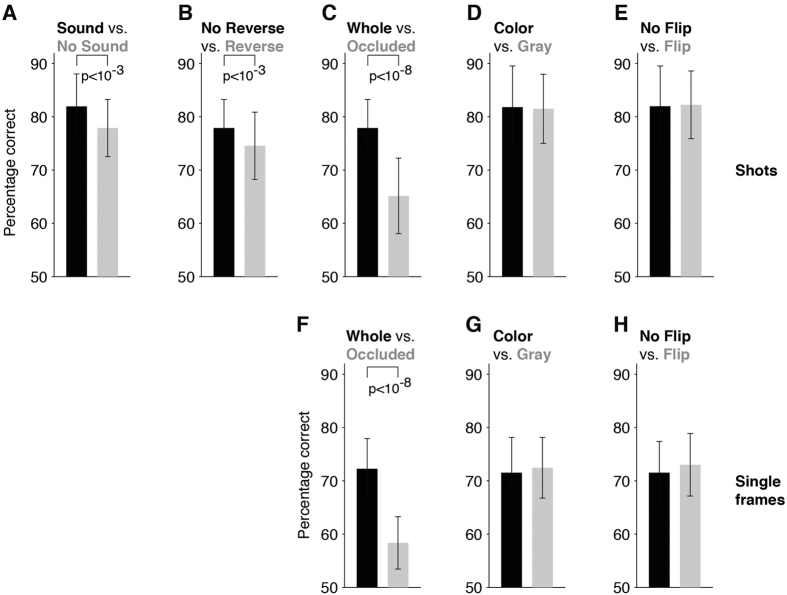
Performance was insensitive to low-level stimulus manipulations and sensitive to disruption of the spatiotemporal events (Variant 4). (**A**) Performance was higher for shots including sounds (black) versus shots where sound was removed (gray). Here and in subsequent plots, the p value shows the results of a non-parametric permutation test (Methods). (**B**) Reversing the temporal order of the frames in a shot (gray) led to decreased performance (here shots did not include sounds). (**C**) Occluding 75% of the frames in a shot (gray) led to decreased performance (here shots do not include sounds). (**E**) Horizontally flipping the frames in a shot (gray) did not change performance. (**D**) Removing color from the frames in a shot (gray) did not change performance. (**F–H**) Same as (**C–E**) but considering only single frames.

In the Main experiment as well as in Variants 1 and 2, the shots during the recognition memory tests were identical to those presented during the movie encoding. We conducted a separate experiment (Variant 3) to evaluate how visual, auditory and temporal characteristics of each shot influenced performance. In this experiment variation, shots were modified during the recognition memory tests by removing sound (Fig. S5B) or color (Fig. S5D), flipping the frames horizontally (Fig. S5C), occluding 75% of each frame (Fig. S5E) or reversing the temporal order of the frames (Fig. S5F). Subjects were instructed to indicate whether the events in the shot had taken place during the movie, irrespective of these manipulations. Removing sound during the recognition memory test impaired performance, but visual information alone was sufficient to drive performance well above chance (Fig. 3A). Reversing the temporal order of the frames in a shot also led to decreased performance (Fig. 3B). Occluding 75% of the content of each frame led to a large decrease in performance both for shots (Fig. 3C) and individual frames (Fig. 3F). Yet, performance was slightly, but significantly, above chance even for occluded single frames (58 ± 5%, p < 10^−4^). In other words, even one quarter of a single frame provided sufficient cues to discriminate whether the corresponding event had been seen before or not. In contrast with removing sound, occlusion or reversing the temporal order of frames, two “low-level” manipulations did not affect performance: neither flipping the frames horizontally (Fig. 3D,H) nor removing color information (Fig. 3E,G) led to changes in performance for either movie shots or single frames. In sum, the variables that led to an increase in the number of errors that subjects made when recognizing specific content from brief shots included distortion of temporal sequences, removal of audio-visual content cues, reduced shot duration and the amount of time between encoding and testing.

The consistency, accuracy and malleability of memory shown here are concordant with previous studies of single items and brief narratives. The current results extend previous work to the domain of spatiotemporal episodic sequences present in movies and establish long-term memorability of movie shots as a robust variable that must be explained from the events occurring during encoding. What determines whether a particular episodic event will be retained or forgotten? We sought to determine which aspects of the content of each shot correlated with successful performance. For this purpose, we used a semi-supervised procedure to annotate each shot in terms of low-level audio-visual properties (contrast, color content, sound volume, sound frequency spectrum), high-level audio-visual properties (specific objects, characters, actions and sounds) and other high-level cognitive properties (e.g. emotional content). The subjects that were involved in these annotations did not participate in any of the memory experiments. An example of these annotations showing the presence (and viewpoint) of each character across the entire first episode is shown in Fig. S6; Tables S2 and S3 list all the content properties that we consider here.

**Figure 4:**
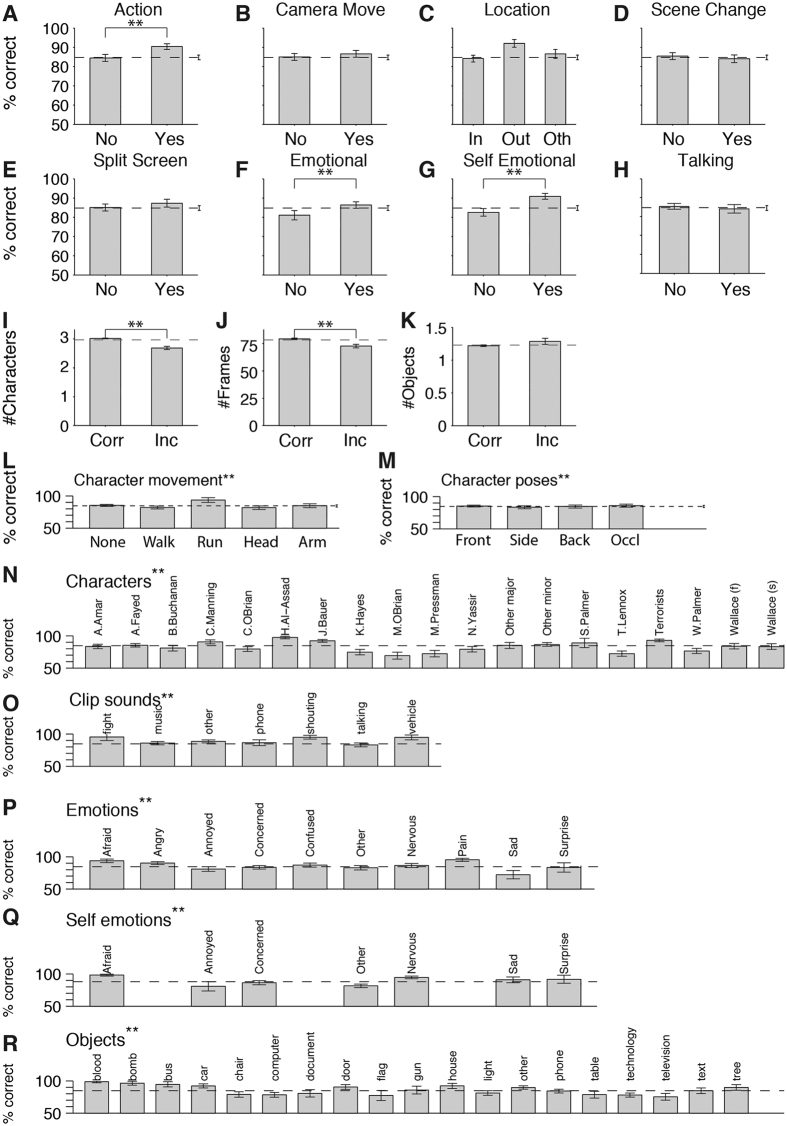
Shot content properties correlated with performance. (**A–H**, **L–R**) Performance for movie shots depending on whether the corresponding content was present or not in the shot (mean ± SEM across subjects). For example, subplot (**A**) indicates the percentage correct in shots where there was no action (“No”) compared to those shots where there was action (“Yes”). The content was manually annotated for each shot by an independent set of subjects who did not participate in the recognition memory study. The definition of each of the content variables is described in the Methods section (see also Tables S2 and S3). (**I–K**) Mean ± SEM values for the number of characters (**I**), number of frames (**J**) and number of objects (**K**) for shots with correct performance (Corr) versus incorrect performance (Inc). In all subplots, “**” denotes p < 0.01 (Bonferroni corrected permutation test).

Several of the annotated content properties showed a significant correlation with performance (Fig. 4). For example, subjects demonstrated enhanced performance in shots containing “action” (90.2 ± 4.6% correct) versus shots without action (84.2 ± 5.9% correct) (Fig. 4A, permutation test *p* = 8 × 10^−7^). Shot content properties that correlated with performance included whether the characters were depicting emotions (Fig. 4F), whether the shot elicited emotions in the viewers (Fig. 4G), the shot duration (Fig. 4J, see also Fig. 2B), the presence of specific characters (Fig. 4N), their poses and movements (Fig. 4L,M), sounds (Fig. 4O), specific emotions (Fig. 4P,Q) and the presence of specific objects (Fig. 4R). By contrast, other variables such as the number of objects, number of characters or camera movement did not correlate with performance (Fig. 4, Tables S2 and S3).

**Figure 5:**
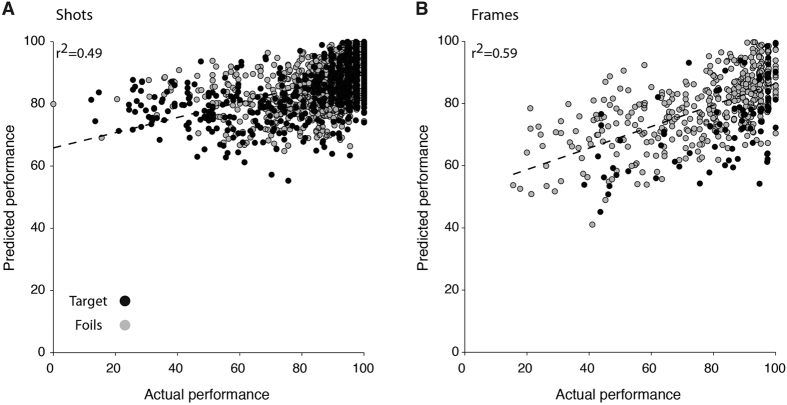
A multivariate linear regression model accounts for a significant fraction of the variance in performance. Multivariate cross-validated linear regression prediction of performance (y-axis) against actual performance (x-axis, the percentage of subjects that was correct) for shots (**A**) or single frames (**B**) for Target (filled circles) or Foils (empty circles). The dashed lines denote the best linear fits. The squared correlation coefficient (*r*^*2*^) is indicated in each subplot.

Inspired by these correlations, we asked whether it was possible to build a simple quantitative model to explain recognition memory performance based exclusively on the content properties. First, we considered a multivariate linear regression model whereby the average performance was described as a linear combination of the content properties (Methods). On average, this multivariate linear regression model was able to account for the degree of memorability in shots (Fig. 5A) as well as in single frames (Fig. 5B) for both episodes (filled and empty circles in Fig. 5). This model accounted for 49% of the variance in the case of shots and 59% of the variance in the case of single frames. The separate contribution of each content variable to this model is shown in Fig. S7.

**Figure 6:**
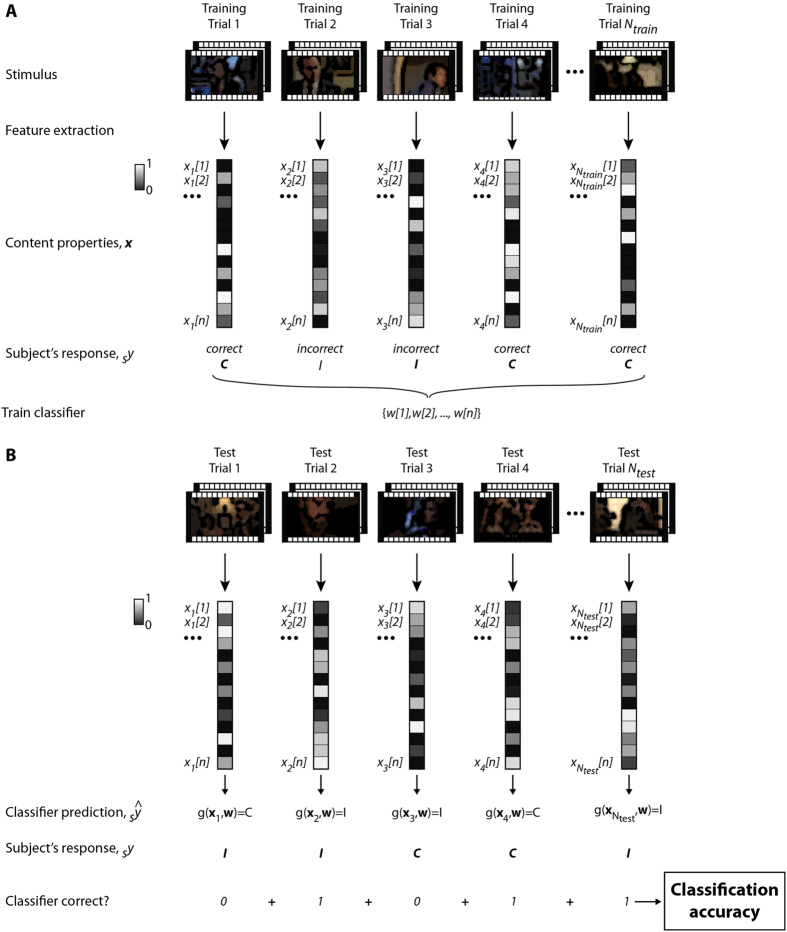
Schematic illustration of the machine learning approach to predict memorability in single trials. The data were randomly divided into a training set with *N*_*train*_ trials (**A**) and a test set with *N*_*test*_ trials (**B**). In each trial, a shot was presented and the subject responded correctly (*C*) or incorrectly (*I*). We extracted the set of *n* content properties *x[1]*, …, *x[n]* for the shot including low-level visual/auditory properties, high-level properties, emotional properties (Methods; Tables S2 and S3). The same approach is followed for single frames. A support-vector machine with a linear kernel was trained to learn the map between the content properties ***x*** and the correct/incorrect labels *y*, resulting in a set of weights *w[1], …, w[n]*. During testing (**B**), we used a different set of shots that did not overlap with the ones in the training set and used the weights w to predict whether the subject was correct or not. By comparing the machine learning predictions with the actual subject responses, we determined whether the classifier was correct or not in each trial and computed the overall classification accuracy (where 50% is chance and 100% is perfect performance). This classification accuracy is shown in Figs 7 and S8–S10).

**Figure 7:**
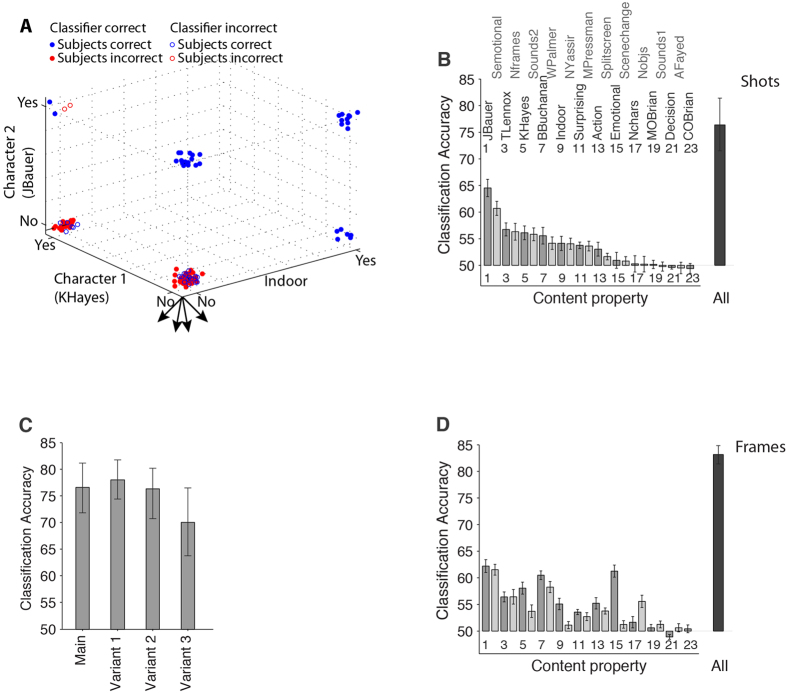
A machine learning classifier achieves high accuracy in predicting performance in single trials. (**A**) Example showing three content properties used to predict memorability in shots where the classifier was correct (filled circles) or incorrect (open circles) for trials where subjects’ performance was correct (blue) or incorrect (red). (**B**) Classifier performance using 23 individual content properties (bars) or combining all properties (“ALL”) for movie shots. Chance performance is 50%. Bars are alternately shown in dark and light gray for aesthetic reasons to better separate them and link them to their labels on top. Odd numbers are also included to link each bar to the appropriate label. (**C**) Classifier performance combining all properties for each experiment variant. (**D**) Same as (**B)** but using individual frames instead of shots.

Building on this linear regression model, we next developed a machine-learning algorithm to predict whether subjects would be correct or incorrect *for each shot or frame*. This model is schematically illustrated in Figs 6 and 7A (Methods). Essentially, the content annotations represent a high-dimensional description of each shot (illustrated in Fig. 7A with only 3 dimensions) and the goal of the algorithm is to find a suitable surface that will separate those shots or frames for which subjects responded correctly from those where they were wrong. We randomly separated all the shots into a training set and a test set to evaluate whether the algorithm trained on one set of shots could extrapolate to a different set (cross-validation). For each shot, we defined a vector ***x*** containing all the content properties (Fig. 4, Tables S2 and S3). Each shot was associated with a label *y* indicating whether subjects performed correctly or incorrectly (C or I, binary classification, either at the group or individual level as described below). During the training phase, we used a support vector machine (SVM) classifier with a linear kernel to learn the map between the content properties ***x*** and the labels *y* (Fig. 6A). To evaluate the performance of this model, we considered different shots not used for training and used the classifier to predict whether subjects would perform correctly or not (Fig. 6B). The shots were randomly subsampled to ensure that chance performance was 50% (otherwise, given that subject performance was above chance levels, the classifier could achieve high accuracy by merely predicting that subjects were always correct). By comparing the classifier predictions with the actual subject responses, we evaluated the classification accuracy, which ranges from 50% (chance) to 100% (perfect predictions). The goal of the classifier was to predict subjects’ performance on a moment-by-moment basis. Hence, the classifier could be correct even when subjects were not and vice versa (e.g. in the example test trial number 2 in Fig. 6B, the subject was incorrect and the classifier correctly predicted this incorrect behavioral response).

First, we considered the group level performance by using the majority vote across subjects as a label for each shot or frame (similar results were obtained when we used the mean response across subjects instead of the majority vote) and training the SVM algorithm to predict performance from individual content properties or combinations of content properties (Methods). In accordance with the correlations for individual content properties described in Fig. 4, there was a wide variation in the classification accuracies from individual content properties (Fig. 7B). Some properties yielded above chance performance (e.g. presence or absence of the main characters in the movie) whereas other properties yielded chance performance (e.g. the number of objects in the shot). Interestingly, the model captured non-trivial properties that relate to the narrative; for example, the individual property that yielded the highest classification accuracy was the presence or absence of the main character in the movie (Jack Bauer, first bar in Fig. 7B). It should be noted that the subsampling procedure ensures that the frequency of occurrence of each content property across the entire movie does not have discriminative power to predict the subjects’ behavior (e.g., the classification accuracy for the main character cannot be simply accounted for by the fact that this character appeared more often than others).

In addition to examining individual contents, the machine-learning algorithm enables us to combine all content properties to make predictions in single shots. When combining all content properties, the classifier performance reached 76.5 ± 4.4% (Fig. 7B). The classifier was even more accurate in predicting subjects’ performance in single frames (83.2 ± 2.5%, Fig. 7D). While there was a positive correlation between the classification accuracy from individual content properties in shots and single frames (compare Fig. 7B versus 7D), some properties were more informative to predict subjects’ performance in shots and other properties were more informative to predict subjects’ performance in single frames. There was a positive correlation in the classification accuracy from each individual property in predicting subjects’ performance for Episode 1 versus Episode 2, but there were also notable differences where properties were more informative to correctly discriminate the target shots in one episode than to correctly rule out the foils in the other episode (Fig. S8). Similar results were obtained when considering data in the 3 experiment variants (Fig. 7C). Furthermore, the properties that contributed most to the classifier performance in one experiment also showed strong contributions in other experiments (Fig. S10). The strongest such correlation was between the Main experiment and Variant 1 (Fig. S10A, *r*^*2*^ = 0.83), where both experiments were identical except that the foil shots in the Main experiment corresponded to the target shots in Variant 1.

The results presented in the previous paragraph describe how well the machine learning approach can predict the *mode*, i.e., the majority vote, across subjects for each shot or frame. Next, we asked whether the classifier could also correctly predict performance for each individual subject. Across individual subjects, the classifier achieved an accuracy of 72.1 ± 10.2% for shots and 63.1 ± 8.3% for single frames (Fig. S9A,B). In order to interpret these classification accuracy values for individual subjects, we considered two simple models that were purely based on behavioral data and not on content properties. In these models, we used subjects’ performance to make predictions within or between subjects. In the first such model (majority-based predictions), we evaluated whether we could predict performance for a given subject from the majority vote of all *other* subjects on the same query shots (Fig. S9C,D, dotted lines; shots: 87.8 ± 5.9%, single frames: 77.7 ± 6.1%).

In the second such model (self-predictions), we evaluated whether we could predict performance in single shots by extrapolating from repeated queries in the same subject (Fig. S9, dash-dotted lines; shots: 85.6 ± 13.3%, single frames: 86.3 ± 7.15%). As expected, both of these models based on human behavior significantly outperformed the machine learning algorithms based on content. We reasoned that these models provide an upper bound for how well any computational algorithm could predict human behavior.

## Discussion

Most input information impinging on our senses is forgotten. The computations involved in specifying which events are to be remembered involve selective and constructive filtering processes to extract meaning based on prior knowledge, goals, associations, and abstraction^1^,^2^,^4^. Here we demonstrate that a computational algorithm can be trained to capture a glimpse of these complex cognitive filtering operations using only visual, auditory and emotional content. Even though this computational algorithm uses only a small fraction of the information present in the inputs, it yields semi-accurate predictions about what moment-by-moment episodic events subjects remember from a movie.

Movies offer the opportunity to examine memory formation for event sequences that are close to the basic elements of everyday episodic recollections. Subjects can form memories for specific movie events that are accurate (Fig. 2A), sufficiently robust to be reproducible across repeated testing (Fig. S4) and yet consistently fallible (e.g. Figs 1C and S2–S4), thus following the basic properties of episodic memory formation demonstrated in other domains^2^,^4^,^5^,^10^. Robust performance was observed across a wide range of conditions including two different movie episodes (Episode 1 in the Main experiment and Episode 2 in Variant 1), when repeatedly testing the same subjects (Main experiments, Variant 1) or when evaluating the performance of each subject only once (Variant 2), for brief shot durations, even for single frames (Fig. 2B) and up to one year post-encoding (Fig. 2C,D).

The observation that recollections are consistent across subjects (Figs 1C and S3–S5) suggests that there are specific aspects of the content of each shot (as well as cultural conventions and similarities) that contribute to remembering and forgetting. The current study provides a quantitative and systematic documentation of how properties of the audio, visual and cognitive contents of brief movie shots contribute to memory formation (Figs 3,4 and S8). The prevalence of certain specific content features is consistent with previous work in the field. For example, a large body of work has linked emotions and memory formation (e.g. refs 16,30, 31, 32, 33, 34); indeed, in our data, the emotional valence of each shot and the emotions elicited in the viewer show a significant correlation with recognition memory performance.

Those content properties can be used in simple models to make single shot predictions of what subjects will or will not remember from specific events embedded within a movie narrative (Figs 5,7 and S9). The proposed model does not aim to capture the mechanisms by which neurons in the brain learn and store these memories but rather provides a quantitative description of how visual, auditory and cognitive variables dictate successful memory encoding.

Recently, some investigators have elegantly used algorithms similar to the ones in the current study to determine what makes individual images memorable (e.g. refs 12 and 35). These studies combined high-throughput behavioral measurements obtained via the web to measure memorability over short temporal scales for isolated images devoid of spatial, temporal or narrative context. The degree of memorability across subjects in those studies could also be predicted from variables describing the contents of each picture. For example, if an image contained faces, it was more likely to be remembered. The work presented here significantly extends those observations in several ways. The content that we study here is dictated by meaningful events that take place during the movie; for example, it is not just any face that drives memorability in our data but specific persons that are relevant to the plot. Here we predict memorability on time scales of weeks to months, up to a year post-encoding, as opposed to the web-based testing of individual items on temporal scales of minutes. We also consider foils that are very similar to the test items in terms of basic properties. Additionally, here we make predictions about memory formation for episodic events that include spatiotemporal context and emotional valence embedded in a narrative as opposed to single items.

How accurate is the model proposed here? To provide an intuition and put the model’s performance in context, we compared it against two models based purely behavioral performance. We reasoned that data from the same individual or the majority across a large number of individuals would constitute a better predictor of a given subject’s behavior than an algorithm that utilizes only a partial description of a shot. Indeed, these alternative models based on human behavior provide upper bounds for classification accuracy and significantly outperform the computational algorithm (Fig. S9). Yet, the machine learning classifier algorithm’s performance was clearly above chance and demonstrated significant explanatory power even for individual subjects. The success of this type of approach is quite remarkable, considering that: (i) only a single shot is used by the algorithm as opposed to subjects who can form associations across the entire narrative of the movie, (ii) a small fraction of the contents is used by the algorithm as opposed to humans who have access to a much richer set of data compared to the rudimentary list of properties in Tables S2 and S3 and (iii) those contents are forced into a rather impoverished format amenable for machine learning classification (Tables S2 and S3). In spite of these limitations, the computational algorithm was only ~10% below the upper bounds provided by the alternative human-based predictions. The proof-of-principle results shown here leave ample room for improvement (e.g. via the incorporation of additional and more accurate content descriptors) while capturing non-trivial aspects of human memory formation.

Even though using movies provides a rich arena to quantitatively examine the formation of episodic memories^22^,^36^,^37^, commercial movies such as the ones used here constitute artificial stimuli where the movie director attempts to guide and manipulate the observers’ viewpoint, attention, feelings and even recollections. Hence, the extent to which the conclusions about the predictability of episodic memory formation from audio, visual and cognitive content can be extrapolated to real life episodic memories remains to be determined and will require further investigation. The initial steps presented here provide a methodological approach that opens the doors to build more complex quantitative models to capture the output of the selective filtering and subjective constructive process that forms the essence of episodic memories.

## Materials and Methods

### Subjects

A total of 161 subjects participated in the main experiment and three variants (Table 1). More than 90% of the subjects were college students or recent graduates. All tests were performed with the subjects’ consent and followed the protocols approved by the Institution Review Board.

### Movie presentation and eye tracking

Subjects watched a 42-minute movie (TV series “24”, Season 6, Episode 1) in the laboratory. None of the subjects had watched any episode from this TV series before. Subjects were instructed to “sit down, relax and enjoy the movie”. During recruitment, subjects were told: “You will be asked questions about the movie in six evaluation sessions” (except in Variant 4, see below). There was no explicit mention about studying or testing memory but it can be surmised that subjects inferred that memory was involved by virtue of the fact that they were going to be asked questions about the movie.

A total of 9 subjects were excluded from analyses: 5 of them were authors in this study and were not considered further to eliminate any potential biases; one subject had a low number of trials (<400), two subjects showed significant biases in the responses (>75% “yes” answers), and one of them had low overall performance (<60% overall). None of the conclusions in the study would be altered if these 9 subjects were included in the analyses. All analyses in the text are based on 152 subjects (Table 1 shows the distribution of these subjects across the four experiments).

The movie was presented on a Sony Multiscan G520 21-inch cathode-ray tube monitor (Sony Corporation, Tokyo, Japan). The movie presentation was controlled by an Apple MacBook Pro computer (Apple Computer, Cupertino, California), using MATLAB software (MathWorks, Natick, Massachusetts) with the Psychophysics Toolbox and Eyelink Toolbox extensions^38^,^39^,^40^. The movie subtended approximately 7.5 × 12.5 degrees of visual angle and was presented in color at 30 frames/sec (multiple figures in the manuscript show examples of movie frames). The audio was delivered via headphones and subjects were allowed to adjust the volume at will. Eye movements were monitored throughout the movie using infrared corneal reflection and pupil location, with nine-point calibration (Eyelink D1000, SR Research, Mississauga, Ontario; there were no “recalibrations” during the movie presentation but accurate calibration was monitored at the end of the movie). Eye tracking data were synchronized to the movie presentation; example eye position data are shown in Fig. 1A. The eye movement data were not used in any of the prediction algorithms.

In those figures that include a frame from the commercial movies (Figs 1A,B,6,S1,S3 and S5), we have replaced the actual original images by artistic renderings.

### Definition of movie shots and content annotation

The sequence of frames during the movie was split into *shots* defined using a computational algorithm to detect sharp transitions (*cuts*) between two consecutive frames (e.g. Fig. S1A). The content of all the movie shots was described using a semi-supervised procedure that included computational annotations and manual annotation by 10 subjects. There was no overlap between the subjects performing these content annotations and those subjects who participated in the recognition memory experiment. The annotations included “low-level” audio and visual properties: contrast, color content, sound level, and sound frequency spectrum. The annotations also included a series of “high-level” properties described in Tables S2 and S3. These properties included whether the shot depicted emotional content, whether the shot elicited emotions in the viewer, whether the shot happened indoors or outdoors, presence or absence of each one of 29 different characters (Fig. S6), viewpoint for each character, presence or absence of 13 possible sounds, presence or absence of 20 possible emotions, and the presence or absence of 25 different objects. Although there was a small degree of variability in the content annotations (particularly for the more subjective aspects of the shot content such as which emotion a character conveyed in a given shot), overall, there was a significant degree of consistency. We used the mode (majority vote) across different annotators when the annotations disagreed. We only considered content properties that appeared in at least 10 shots for analyses. An example of these annotations is shown in Fig. S6.

### Foil shots

Recognition memory evaluation sessions included shots from Episode 1 (the episode that subjects watched, referred to as “Target” throughout the manuscript) and Episode 2 of the same TV series and season (not watched by the subjects, referred to as “Foil” throughout the manuscript). Targets and foils are counterbalanced in Variant 1, described below. The selection of suitable foils is critical in memory experiments. For example, the task can be trivial if the foils are taken from a cartoon movie and the task can be made virtually impossible if only one pixel in the entire frame is changed. The task was specifically designed to include a natural comparison of targets and foils that would resemble the formation of episodic memories in realistic scenarios. The events in a given season of this TV series take place during a twenty-four hour period; this means that when comparing two consecutive episodes, each character is typically wearing the same clothes, the locations are similar, the filming style is the same, etc. To further ensure that targets and foils were similar, (i) we matched the average shot duration in target and foils, and (ii) we selected shots from Episode 1 that had a corresponding shot in Episode 2 that was matched as close as possible in terms of the content annotations for characters and their viewpoints. Examples of such matches across episodes are provided in Fig. S1B. For every target shot shown from Episode 1, there was a trial with a matching foil shot from Episode 2 containing the same characters and viewpoints.

### Recognition memory evaluation

In each trial, subjects were presented with either a target or a foil shot. Shots from either episode were shown in pseudo-random order and with equal probability (chance performance was 50%). Subjects performed an old/new task reporting whether they remembered having seen the events in the shot during the movie presentation or not (Fig. 1B). Responses were provided using a computer mouse.

There were four different experiments. Performance in each variant is shown in Table S1. Throughout the text, we focus on the Main experiment unless otherwise stated.

#### Main experiment

Performance was evaluated in six sessions: Session 1, immediately after watching the movie (referred to as 0 days); Session 2, between 22 and 26 hours after watching the movie (referred to as 1 day); Session 3, between day 6 and day 8 after watching the movie (referred to as 7 days); Session 4, between day 27 and day 33 after watching the movie (referred to as 30 days); Session 5, between 85 and 95 days after watching the movie (referred to as 90 days); Session 6, between 335 and 395 days after watching the movie (referred to as 365 days). Subjects were offered a monetary incentive that grew with the number of sessions in which they participated. Still, not all subjects finished all 6 sessions (average 3.7 ± 1.1 sessions/subject). The dependence of performance with the time between encoding and testing is described in Fig. 2C,D. Subjects were instructed not to watch any episode of this TV series during the entire testing period of 365 days. All subjects reported compliance with this rule.

In order to evaluate self-consistency (Figs 1C and S6), unbeknown to the subjects, a small fraction (3%) of the shots was repeated at random times during the test. These repeat trials were equally distributed between the main movie and the control. None of the conclusions would be altered if these trials were excluded from the analyses (except of course that we would not be able to report self-consistency). There was no systematic trend in performance when comparing the first presentation of each shot and subsequent repetitions for this small set of 3% of repeated trials.

#### Variant 1

In this experiment variant, the role of Episode 1 and Episode 2 were reversed. Subjects watched Episode 2 during the movie encoding session and foil shots were taken from Episode 1. All other procedures were identical to the Main experiment.

#### Variant 2

In this experiment variant, recognition memory was only evaluated in one session for each subject. All other procedures were identical to the Main experiment.

#### Variant 3

We refer to the presentation of unaltered shots as the default condition (Fig. S5A). In this experiment variant, a series of modifications of each shot were introduced during the recognition memory test sessions: (i) presentation of single frames (randomly chosen from within the test shots); (ii) removal of sound (Fig. S5B); (iii) horizontal flip of each frame from left to right (Fig. S5C); (iv) grayscale presentation (Fig. S5D); (v) occlusion, by presenting only one quadrant (randomly selected) and covering the other three quadrants with a black occluder (Fig. S5E); (v) temporal reversal of the frames within the shot (Fig. S5F). Subjects were instructed to indicate whether they remembered the events depicted in the shot regardless of such transformations. The order of presentation of shots and these manipulations was pseudo-randomized. All other procedures were identical to the Main experiment.

### Data analyses

We computed the total number of “yes” and “no” responses for each subject. With the exception of one subject who was excluded from analyses (discussed above), the proportion of yes and no responses was close to 50% (50.5 ± 4.9%, mean ± SD across subjects).

Throughout the manuscript, we summarized performance for each experimental condition by reporting the percentage of trials in which subjects were correct (*pc*, “percentage correct”). The overall percentage of correct trials combines the probability of hits (*p*_*hit*_, the probability of reporting a correct answer when the target was shown) and the probability of false alarms (*p*_*FA*_, the probability of reporting an incorrect answer when the foil was shown). Given that the number of target and foil trials was approximately the same, 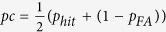. It is also common to combine *p*_*hit*_ and *p*_*FA*_ by reporting d′, 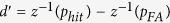, where *z*^*−1*^(*p*) indicates the z score corresponding to the probability *p*^41^. There were no significant biases and the performance in target trials was comparable to the performance in foil trials (Fig. 2A, Table S1). We report *p*_*hit*_, *p*_*FA*_ and *d’* in Table S1 and in supplementary web figures that match Figs 2 and 3 in the main text (http://klab.tch.harvard.edu/resources/Tangetal_episodicmemory_2016.html). None of the conclusions in this study change if we use these alternative metrics and we opted to consistently keep one metric, the overall percentage correct, throughout the text rather than reporting multiple different values for each figure.

The first 5 trials in each experimental recognition memory session were removed from analyses to avoid any non-stationarities while subjects were adapting to the test. Because each subject participated in over a thousand trials (mean = 1629 trials per subject), removing these 5 trials did not affect the results. Throughout the manuscript and unless otherwise stated, statistical analyses are based on a two-sided non-parametric permutation test with Bonferroni correction^42^. We only computed percentages for a given condition if we had a minimum of 20 trials.

When evaluating the degree of consistency, within and across subjects, we compared results against the null hypothesis according to which performance was independent across trials. Let *p*_*i*_ be the percentage correct for subject *i*. Under the independence assumption, we expect the fraction of repeat trials when subject *i* is consistently correct to be 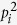, the fraction of repeat trials when subject i is consistently wrong to be (1−*p*_*i*_)^2^ and the fraction of repeat trials when subject is inconsistent to be 2*p*_*i*_(1−*p*_*i*_) (where the factor 2 arises because of the two possible ways of being inconsistent; note that 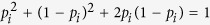). Similarly, when considering two subjects *i* and *j*, under the null hypothesis, the fraction of repeat trials when both subjects are expected to be correct is *p*_*i*_*p*_*j*_, the fraction of repeat trials when both subjects are expected to be wrong is (1−*p*_*i*_)(1−*p*_*j*_) and the fraction of repeat trials when the subjects are expected to be inconsistent is... The results of simulations to evaluate these expected values under the null hypothesis are shown in Fig. S4.

We evaluated whether the content of each shot (see “*Definition of movie shots and content annotation”* and Tables S2 and S3) correlated with behavioral performance. Let the vector ***x***_*i*_ denote the content of shot *i*. The dimensionality of this vector (dim(***x***)) depended on which content properties were used for the analyses (a schematic rendering of the feature extraction process is shown in Fig. 6; an example using three properties is shown in Fig. 5A). Let _*s*_*y*_*i*_ indicate whether subject *s* was correct or not in shot *i* 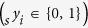. We also considered the response mode (majority vote) across subjects, 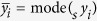. We examined the correlation between ***x*** and *y* for each individual content property (Fig. S7). We also considered a multivariate linear regression model defined by 
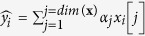
 where the coefficients α_*j*_ were fit to the data Fig. 5).

We quantitatively evaluated how well we could predict subjects’ recognition memory performance using the properties describing the content of each shot on individual trials (Figs 6,7, S8 and S9). A schematic description of the machine learning approach is shown in Fig. 6. We used a machine learning approach to learn the map between ***x***_*i*_ and the recognition memory performance of each subject, _*s*_*y*_*i*_^43^,^44^. We also considered the response mode across subjects 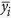 and evaluated whether we could predict this majority vote (binary yes/no decision) for each shot. We used a Support Vector Machine (SVM) classifier with a linear kernel: the algorithm’s boundary can be described by **w.x**_*i*_ where **w** are weights that are adjusted during training. We used a ten-fold cross-validation approach to avoid overfitting. To ensure that chance performance was 50% for the algorithm, we randomly subsampled the data such that #{*y*_*i*_ = 1}=#{*y*_*i*_ = 0}. Several other algorithms were also evaluated: Fisher linear discriminant classifier, a nearest neighbor classifier, a naïve Bayesian classifier. While the exact performance value showed a small dependence on the machine-learning algorithm used, none of the conclusions depended on the algorithm choice. In the interest of simplicity, we report results for only one algorithm (SVM, which is known to show robust generalization performance). To evaluate the expected performance under the null hypothesis that there is no correlation between the movie content and recognition memory performance, we randomly shuffled the shots and recomputed the classification performance (10,000 iterations).

### Ethical approval and informed consent

All experimental protocols were approved by the Institutional Review Board at Children’s Hospital. All the methods were carried out in accordance with the approved guidelines. Informed consent was obtained from all subjects.

## Additional Information

**How to cite this article**: Tang, H. *et al*. Predicting episodic memory formation for movie events. *Sci. Rep.* **6**, 30175; doi: 10.1038/srep30175 (2016).

## Supplementary Material

Supplementary Information

## Figures and Tables

**Table 1:** Summary of number of subjects and test conditions for each variation of the experiment.

	MAIN	VARIANT 1	VARIANT 2	VARIANT 3
Number of subjects	41 (41)	24 (22)	39 (37)	57 (52)
Number of subjects tested at 1 year	18	0	4	20
Age range	18–48	18–48	18–39	20–28
Age mean ± SD	24.8 ± 7.0	28.5 ± 9.7	23.0 ± 4.7	23.9 ± 1.7
Percentage female	49	54	44	60
Encoding episode	1	2	1	1
Recognition memory tested in one session only	No	No	Yes	No
Stimulus transformations during recognition memory test	No	No	No	Yes

The main experiment and variants are described in the main text and in the Methods section. The number of subjects indicates the total number of participants and, in parenthesis, the number of participants that were included in the analyses (see exclusion criteria in the Methods section). Performance in each experiment variant is shown in Table S1.
