# Supplementary material for: Predicting episodic memory formation for movie events
Source: Sci Rep. 2016 Sep 30;6:30175. doi: 10.1038/srep30175 (PMC5043190; doi:10.1038/srep30175)
Supplement: Supplementary Information [file srep30175-s1.pdf]

Predicting episodic memory formation for movie events

Hanlin Tang[1,4], Jed Singer[1], Matias Ison[1], Gnel Pivazyan[2], Melissa Romaine[3], Rosa Frias[1], Elizabeth Meller[1], Adrianna Boulin[3], James Carroll[3], Victoria Perron[1], Sarah Dowcett[3], Marlise Arellano[1], and Gabriel Kreiman[1,4*]

[1] Children's Hospital, Harvard Medical School
[2] UC Berkeley
[3] Emmanuel College
[4] Program in Biophysics, Harvard University
*Corresponding author: gabriel.kreiman@tch.harvard.edu

List of Supplementary Material

Supplementary Tables: 3

Supplementary Figures: 10

Supplementary Table Legends

Table S1. Summary statistics for recognition memory performance for all experiments

Mean performance across subjects (±SD) is reported separately for shots (columns 1-4) and single frames (columns 5-8). We report P_{hit} (the proportion correct for target trials), P_{FA} (the proportion of false alarms during foil trials), PC (the overall percentage of correct trials including targets and foils) and d'. The number of subjects for each experiment is shown in **Table 1**. Each experiment variant is described in the Methods section.

	**Shots**				**Frames**			
	**P_{hit}**	**P_{FA}**	**PC**	**d'**	**P_{hit}**	**P_{FA}**	**PC**	**d'**
**Main**	0.87 (0.07)	0.16 (0.08)	85.6 (5.3)	2.21 (0.43)	0.79 (0.09)	0.22 (0.09)	78.2 (6.0)	1.62 (0.37)
**Variant 1**	0.77 (0.10)	0.12 (0.09)	82.5 (7.0)	2.03 (0.53)	0.64 (0.12)	0.19 (0.11)	72.1 (6.7)	1.29 (0.39)
**Variant 2**	0.82 (0.08)	0.24 (0.10)	79.2 (5.9)	1.67 (0.41)	0.70 (0.13)	0.28 (0.12)	70.8 (6.5)	1.17 (0.34)
**Variant 3**	0.81 (0.09)	0.22 (0.08)	79.5 (6.3)	1.73 (0.47)	0.74 (0.11)	0.32 (0.12)	70.0 (6.5)	1.12 (0.39)

Table S2. Definition of high-level content annotations (part 1)

Definition and description of content variables (columns 2-3) that were manually annotated for each shot. For each variable we report the annotation reliability (column 4), computed as the average fraction of consistent responses across annotators (maximum = 1.0, chance = 0.50). For variables 1-10, we report statistics for recognition memory performance for each of the two possible annotation values; these statistics including number of shots (columns 7, 11), mean percentage correct across subjects (columns 8, 12), standard deviation of the percentage correct (columns 9, 13) and a permutation test (column 14) evaluating the null hypothesis that recognition memory performance is independent of the variable value. Entries with Bonferroni corrected permutation p values < 0.01 are highlighted in gray. The description of Variables 11-15 is expanded in **Table S3**. For variables 16-18, we report the mean and SD for those shots with correct versus incorrect recognition memory performance. Results shown in this table correspond to the Main experiment.

Table S3. Definition of high-level content annotations (part 2)

Expanding on variables numbered 11 through 15 from **Table S2**, here we enumerate the variables that were manually annotated for each shot describing the presence or absence of specific characters, sounds, objects and emotions. For each case, we report recognition memory performance statistics including the mean % correct, SD % correct and the results of a permutation test evaluating the null hypothesis that recognition memory performance was independent of the presence or absence of that character/sound/object/emotion. Entries with Bonferroni corrected permutation p values < 0.01 are highlighted in gray. Only entries occurring in at least 10 shots are described here and used for analyses. Results shown in this table correspond to the Main experiment.

TABLE S2

ID	Property	Brief definition	Annotation reliability	Type/ Values	Value	N	Mean % correct	SD % correct	Value	N	Mean % correct	SD % correct	Perm. test	Comments
1	Action	Whether shot contains actions (e.g. running, walking, explosions)	0.88	Binary {Y/N}	No	775	84.2	5.9	Yes	206	90.2	4.6	8E-07	
2	Camera movement	Whether camera or zoom moved during the shot	0.78	Binary {Y/N}	No	750	84.8	5.8	Yes	231	86.4	5.6	0.15	
3	Decision	Whether a decision was made during shot	0.93	Binary {Y/N}	No	954	84.3	5.8	Yes	27	84.4	5.4	0.22	
4	Indoor/outdoor/other	Shot is indoors, outdoors or other	0.99	{In, Out, Other}	In	771	83.9	5.6	Out	186	91.9	6.3	4.00E-08	24 shots were labeled as "other", with both indoor and outdoor content
6	Surprising	Whether the events during the shot are surprising to the viewer	0.99	Binary {Y/N}	No	945	84.3	5.8	Yes	36	84.4	5.4	0.43	

7	Scene change	Location change with respect to previous shot	0.99	Binary {Y/N}	No	913	85.2	5.7	Yes	68	83.8	6.2	0.27	
8	Split screen	Director used split screens to convey events in separate locations	0.99	Binary {Y/N}	No	903	84.8	5.7	Yes	78	87.1	6.6	0.008	
9	Emotional	Whether shot conveys emotions	0.83	Binary {Y/N}	No	304	80.8	7.7	Yes	677	86.2	5.3	0.0003	
10	Self emotional	Whether shot elicits emotions in viewer	0.78	Binary {Y/N}	No	634	82.3	6.3	Yes	347	90.7	4.8	3E-09	
11	Objects	Presence or absence of each of 25 specific labeled objects (e.g. chair, computer, etc.)	0.99	Binary {Y/N} (for each object)										See Table S3
12	Characters	Presence or absence of each of 29 characters	0.98	Binary {Y/N} (for each character)										See Table S3

13	Viewpoints	Front/Side/Back/Occluded for each character present in shot	0.98	{F,S,B,O} (for each character)										See Table S3
14	Sounds	Presence or absence of each of 13 specific sounds	0.99	Binary {Y/N} (for each sound)										See Table S3
15	Emotions	Presence or absence of each of 20 specific emotions	0.97	Binary {Y/N} (for each emotion)										See Table S3
**ID**	**Property**	**Brief definition**		**Type/ Values**	**Correct**	**N**	**Mean**	**SD**	**Incorrect**	**N**	**Mean**	**SD**	**Perm. test**	**Comments**
16	Number of frames	Number of frames in shot		Integer [1,...,200]	Corr	838	79.5	54.6	Incorr	143	72.6	49.2	2E-09	
17	Number of objects	Number of main labeled objects		Integer [1,...,25]	Corr	838	1.2	1.4	Incorr	143	1.3	1.5	0.11	
18	Number of characters	Number of characters present in shot		Integer [1,..,29]	Corr	838	3.0	2.2	Incorr	143	2.7	2.0	4E-14	

TABLE S3

Characters	Name	N	Mean % correct	SD % correct	Perm. Test	Only 19 characters (with >10 appearances) out of 29 characters shown here
1	A.Amar	88	83.1	7.4	0.18	
2	A.Fayed	153	85.0	5.9	0.73	
3	B.Buchanan	224	80.5	9.7	0.0088	
4	C.Manning	92	90.7	6.7	1.1E-06	
5	C.OBrian	88	79.3	8.0	0.0001	
6	H.Al-Assad	73	97.4	4.2	5.3E-22	
7	J.Bauer	382	92.1	4.5	3.9E-13	
8	K.Hayes	155	74.1	9.2	5.00E-09	
9	M.OBrian	47	68.8	11.3	3.1E-11	
10	M.Pressman	64	71.8	10.6	1.3E-09	
11	N.Yassir	74	78.8	9.3	0.0002	
12	Other major	23	84.4	9.9	0.85	
13	Other minor	335	86.3	6.0	0.11	
14	S.Palmer	12	88.7	12.2	0.089	
15	T.Lennox	146	71.8	9.1	2.5E-11	
16	Terrorists	152	92.8	4.6	4.70E-14	
17	W.Palmer	178	75.9	8.5	6.9E-08	
18	Wallace (f)	82	83.6	9.1	0.44	
19	Wallace (s)	66	83.0	9.4	0.25	
**Sounds**	**Name**	**N**	**Mean % correct**	**SD % correct**	**Perm. Test**	Only 7 sounds (with >10 appearances) out of 13 sounds shown here
1	fight	30	95.3	11.5	1.1E-06	
2	music	856	85.6	5.6	0.32	
3	other	83	88.5	5.1	2.5E-05	

4	phone	19	86.6	9.1	0.19	
5	shouting	40	94.8	6.0	8.1E-13	
6	talking	580	82.9	6.5	0.079	
7	vehicle	15	94.6	7.8	1.4E-09	
**Emotions**	**Name**	**N**	**Mean % correct**	**SD % correct**	**Perm. Test**	Only 10 emotions (with >10 appearances) out of 20 emotions shown here
1	Afraid	53	93.3	5.6	3E-12	
2	Angry	40	90.0	5.0	4E-08	
3	Annoyed	27	81.0	7.5	0.003	
4	Concerned	331	83.5	5.8	0.19	
5	Confused	45	86.9	6.3	0.03	
6	Other	142	82.8	7.4	0.11	
7	Nervous	34	86.2	6.5	0.16	
8	Pain	29	95.2	5.1	4E-16	
9	Sad	18	71.8	13.7	4E-07	
10	Surprise	16	83.4	13.4	0.6	
**Emotions (self)**	**Name**	**N**	**Mean % correct**	**SD % correct**	**Perm. Test**	Only 7 emotions (with >10 appearances) out of 20 emotions shown here
1	Afraid	21	100.0	0.0	0	
2	Annoyed	13	61.5	17.6	2.00E-05	
3	Concerned	162	85.4	4.7	0.18	
4	Other	132	80.1	5.1	0.0003	
5	Nervous	102	91.1	12.7	0.11	
6	Sad	32	89.9	7.2	0.087	
7	Surprise	13	87.2	5.4	0.3	
**Objects**	**Name**	**N**	**Mean % correct**	**SD % correct**	**Perm. Test**	Only 19 objects (with >10 appearances) out of 25 objects shown here
1	blood	11	98.7	2.9	1E-21	

2	bomb	23	96.3	5.6	3E-13	
3	bus	20	94.5	7.7	9.7E-09	
4	car	56	92.1	6.8	2.2E-08	
5	chair	80	78.4	9.3	0.0001	
6	computer	88	77.9	8.1	2.9E-06	
7	document	29	80.1	12.1	0.02	
8	door	32	90.1	7.8	6.8E-05	
9	flag	24	76.4	16.6	0.0027	
10	gun	20	85.3	12.4	0.78	
11	house	19	92.2	7.3	2.1E-06	
12	light	66	80.9	7.4	0.0022	
13	other	80	89.5	5.4	1.5E-06	
14	phone	136	84.0	6.3	0.47	
15	table	66	78.0	11.3	0.00049	
16	technology	36	77.7	7.0	1.1E-07	
17	television	57	74.9	11.4	2.4E-06	
18	text	30	84.6	9.0	0.96	
19	tree	15	89.7	9.0	0.0027	

TABLE S3

Characters	Name	N	Mean % correct	SD % correct	Perm. Test	Only 19 characters (with >10 appearances) out of 29 characters shown here
1	A.Amar	88	83.1	7.4	0.18	
2	A.Fayed	153	85.0	5.9	0.73	
3	B.Buchanan	224	80.5	9.7	0.0088	
4	C.Manning	92	90.7	6.7	1.1E-06	
5	C.OBrian	88	79.3	8.0	0.0001	
6	H.Al-Assad	73	97.4	4.2	5.3E-22	
7	J.Bauer	382	92.1	4.5	3.9E-13	
8	K.Hayes	155	74.1	9.2	5.00E-09	
9	M.OBrian	47	68.8	11.3	3.1E-11	
10	M.Pressman	64	71.8	10.6	1.3E-09	
11	N.Yassir	74	78.8	9.3	0.0002	
12	Other major	23	84.4	9.9	0.85	
13	Other minor	335	86.3	6.0	0.11	
14	S.Palmer	12	88.7	12.2	0.089	
15	T.Lennox	146	71.8	9.1	2.5E-11	
16	Terrorists	152	92.8	4.6	4.70E-14	
17	W.Palmer	178	75.9	8.5	6.9E-08	
18	Wallace (f)	82	83.6	9.1	0.44	
19	Wallace (s)	66	83.0	9.4	0.25	
**Sounds**	**Name**	**N**	**Mean % correct**	**SD % correct**	**Perm. Test**	Only 7 sounds (with >10 appearances) out of 13 sounds shown here
1	fight	30	95.3	11.5	1.1E-06	
2	music	856	85.6	5.6	0.32	
3	other	83	88.5	5.1	2.5E-05	

4	phone	19	86.6	9.1	0.19	
5	shouting	40	94.8	6.0	8.1E-13	
6	talking	580	82.9	6.5	0.079	
7	vehicle	15	94.6	7.8	1.4E-09	
**Emotions**	**Name**	**N**	**Mean % correct**	**SD % correct**	**Perm. Test**	Only 10 emotions (with >10 appearances) out of 20 emotions shown here
1	Afraid	53	93.3	5.6	3E-12	
2	Angry	40	90.0	5.0	4E-08	
3	Annoyed	27	81.0	7.5	0.003	
4	Concerned	331	83.5	5.8	0.19	
5	Confused	45	86.9	6.3	0.03	
6	Other	142	82.8	7.4	0.11	
7	Nervous	34	86.2	6.5	0.16	
8	Pain	29	95.2	5.1	4E-16	
9	Sad	18	71.8	13.7	4E-07	
10	Surprise	16	83.4	13.4	0.6	
**Emotions (self)**	**Name**	**N**	**Mean % correct**	**SD % correct**	**Perm. Test**	Only 7 emotions (with >10 appearances) out of 20 emotions shown here
1	Afraid	21	100.0	0.0	0	
2	Annoyed	13	61.5	17.6	2.00E-05	
3	Concerned	162	85.4	4.7	0.18	
4	Other	132	80.1	5.1	0.0003	
5	Nervous	102	91.1	12.7	0.11	
6	Sad	32	89.9	7.2	0.087	
7	Surprise	13	87.2	5.4	0.3	
**Objects**	**Name**	**N**	**Mean % correct**	**SD % correct**	**Perm. Test**	Only 19 objects (with >10 appearances) out of 25 objects shown here
1	blood	11	98.7	2.9	1E-21	

2	bomb	23	96.3	5.6	3E-13	
3	bus	20	94.5	7.7	9.7E-09	
4	car	56	92.1	6.8	2.2E-08	
5	chair	80	78.4	9.3	0.0001	
6	computer	88	77.9	8.1	2.9E-06	
7	document	29	80.1	12.1	0.02	
8	door	32	90.1	7.8	6.8E-05	
9	flag	24	76.4	16.6	0.0027	
10	gun	20	85.3	12.4	0.78	
11	house	19	92.2	7.3	2.1E-06	
12	light	66	80.9	7.4	0.0022	
13	other	80	89.5	5.4	1.5E-06	
14	phone	136	84.0	6.3	0.47	
15	table	66	78.0	11.3	0.00049	
16	technology	36	77.7	7.0	1.1E-07	
17	television	57	74.9	11.4	2.4E-06	
18	text	30	84.6	9.0	0.96	
19	tree	15	89.7	9.0	0.0027	

Figure S1

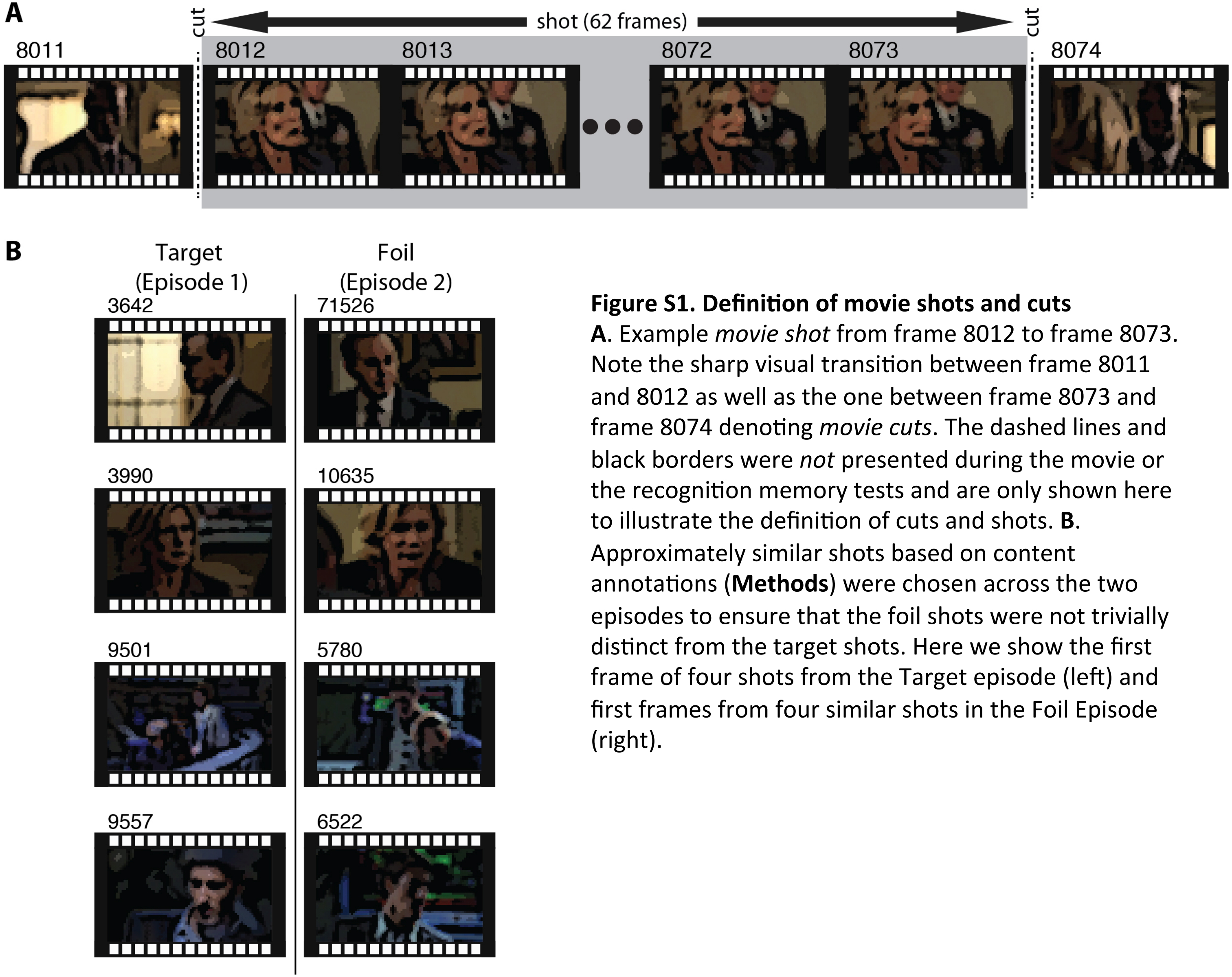

Figure S1. Definition of movie shots and cuts
A. Example *movie shot* from frame 8012 to frame 8073. Note the sharp visual transition between frame 8011 and 8012 as well as the one between frame 8073 and frame 8074 denoting *movie cuts*. The dashed lines and black borders were *not* presented during the movie or the recognition memory tests and are only shown here to illustrate the definition of cuts and shots. **B**. Approximately similar shots based on content annotations (**Methods**) were chosen across the two episodes to ensure that the foil shots were not trivially distinct from the target shots. Here we show the first frame of four shots from the Target episode (left) and first frames from four similar shots in the Foil Episode (right).

Figure S2

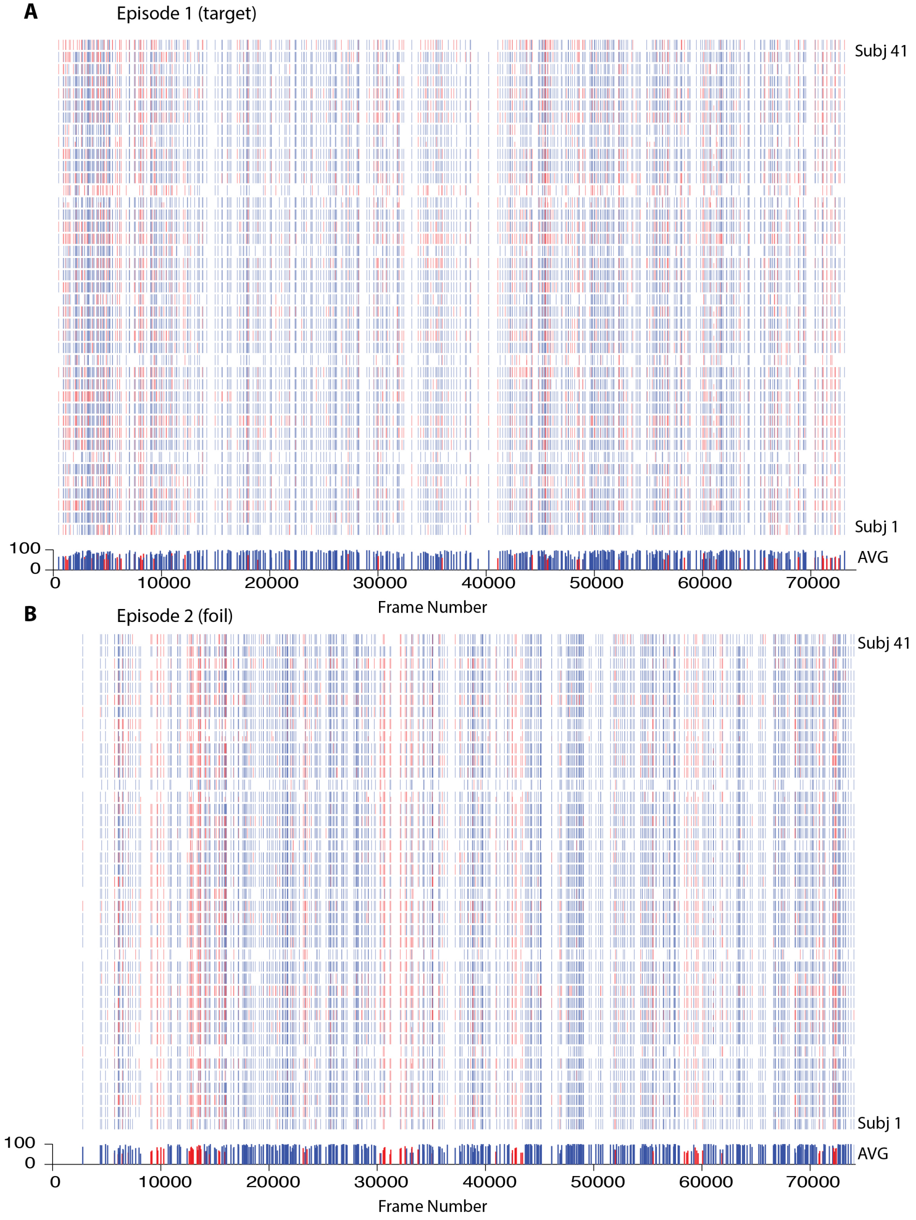

Figure S2. Raster plots showing performance across the movie

Extending on the example segment shown in **Figure 1C**, these raster plots show the performance of all subjects for all shots during target episode (**A**) or foil episode (**B**). The format is the same as that in **Figure 1C**. The dashed box in part **A** corresponds to the movie segment shown in **Figure 1C**. The order of queries during the recognition memory tests was randomized.

Figure S3

A

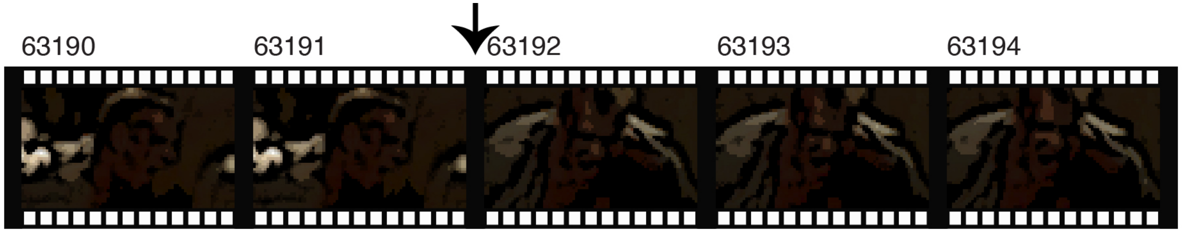

consistent right

n= 41 responses (41Y)
correct answer = Y

YYYYYYYYYYYYY YYYYYYYYYY YYYYYYYYYY YYYYYYYY

B

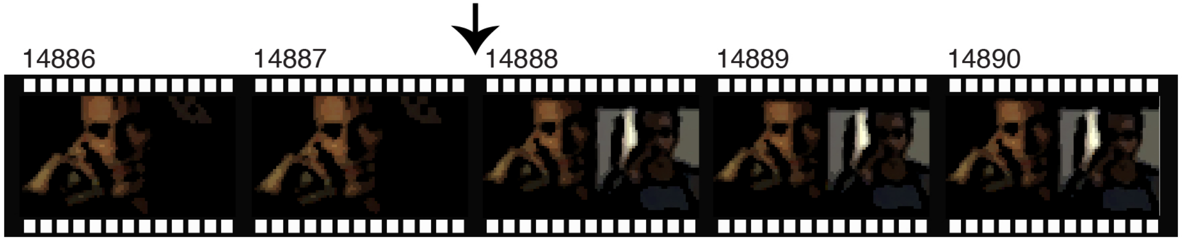

inconsistent

n= 31 responses (18Y)
correct answer = N

NNYYYNNNYYYYYYYYNNNY NNYYNNYYYNY

C

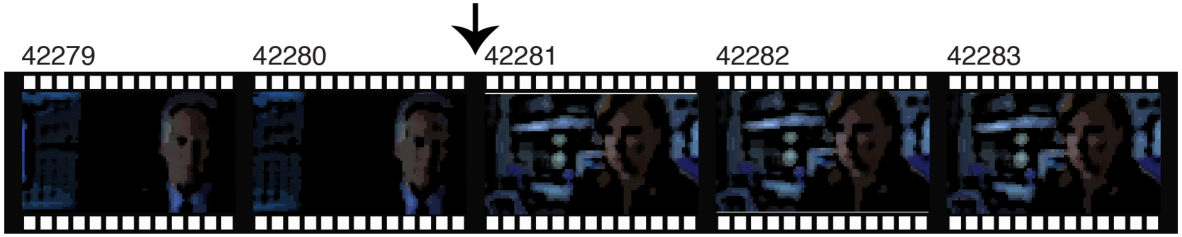

consistent wrong

n=32 responses (29Y)
correct answer = N

NYYYYYYYYYYYNYYYYYYYYYYYYYYYNYYY

Figure S3. Examples of consistent and inconsistent performance across subjects
Three examples showing consistently correct answers (**A**, 41 out 41 answers correct), consistently incorrect answers (**C**, 29 out of 32 answers incorrect) and inconsistent answers (**B**, 18 out 31 answers correct). In each example, we show two frames before and three frames after the cut transition (arrow). We show the correct answer and each individual answer (Y/N).

Figure S4

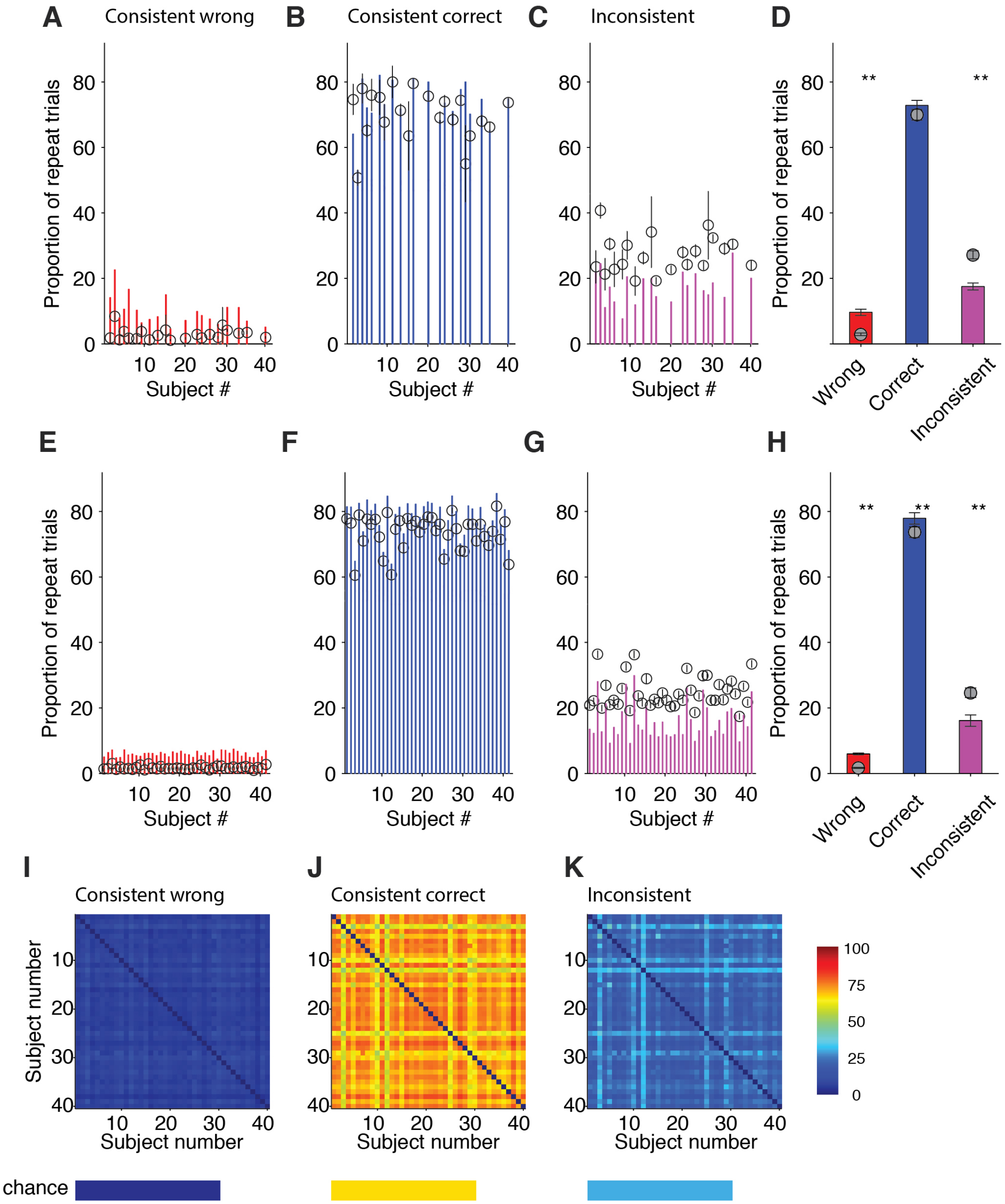

Figure S4. Subjects showed consistent performance
A-D. Degree of self-consistency for each subject (gaps denote subjects without sufficient number of trials for this analysis). We considered repeat trials where the same frame or shot was shown at two random time points during the recognition memory test (**Methods**). Percentage of repeat trials where subjects were consistently incorrect (**A**, red), consistently correct (**B**, blue) or inconsistent (**C**, pink). The circles show the expected performance (±SD) under the null hypothesis that repeat trials are independent after considering the overall percentage correct for each subject (**Methods**). The proportion of consistently correct or incorrect trials was significantly higher than expected under the null hypothesis whereas the proportion of inconsistent trials was significantly lower than expected under the null hypothesis. **D** shows the averages.
E-H. Comparison of each subject's performance against the majority vote of all other subjects (formats and conventions as in part **A-D**).
I-K. Pairwise comparisons reflecting between-subject consistency for repeat trials. Entry (*i*,*j*) in these symmetric matrices denotes the percentage of trials when both subjects *i* and *j* were incorrect (**I**), correct (**J**) or inconsistent (**K**); see color scale bar on right. The expected value under the null hypothesis is shown as a colored bar below each matrix.

Figure S5

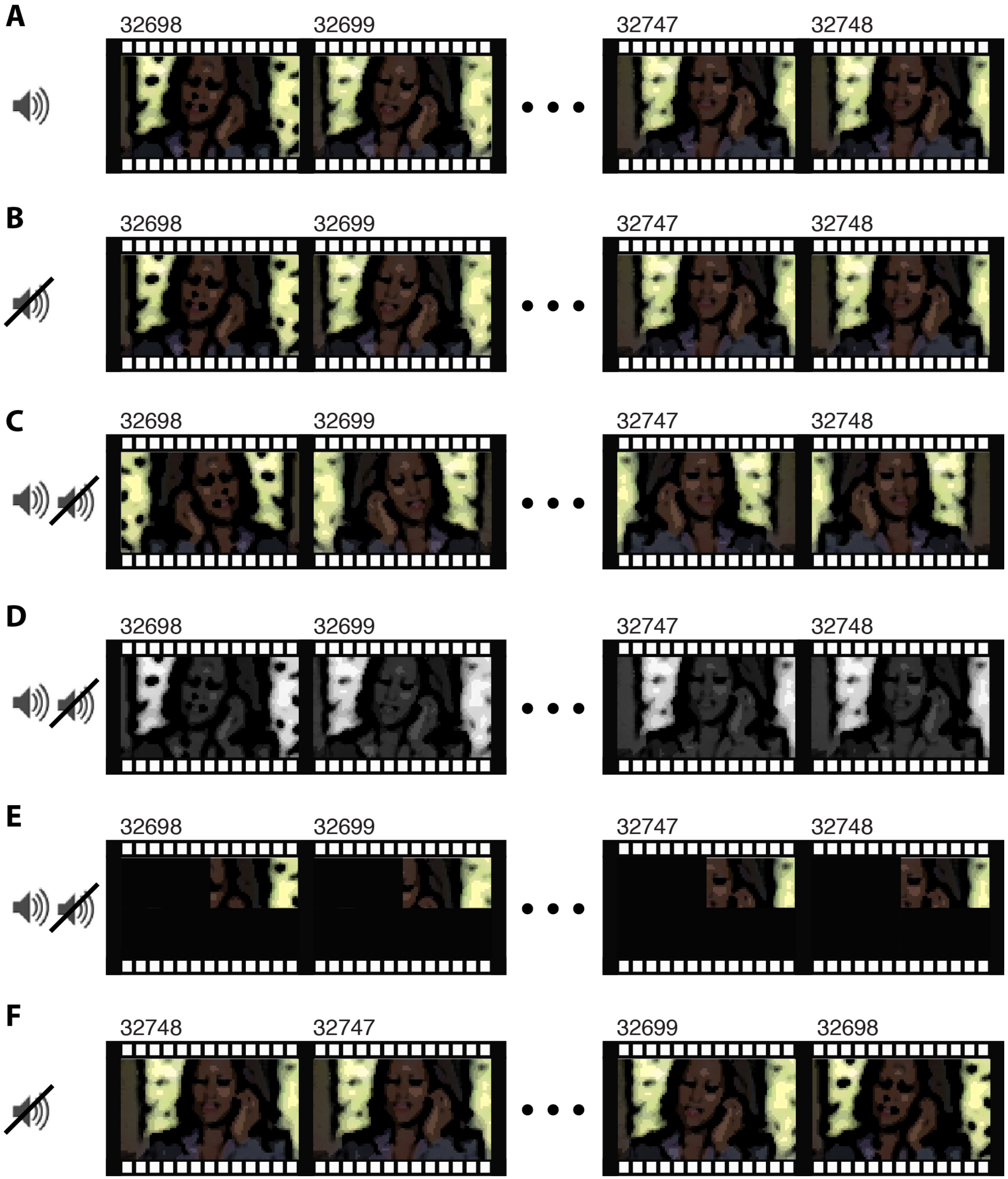

Figure S5. Manipulations examined during the recognition memory test (Variant 3)
A. Example shot from frame 32698 to frame 32748. **B**. Same shot shown without the sound. **C**. Frames horizontally flipped. **D**. Color information removed and frames shown in grayscale. **E**. Occlusion, where 75% of the frame was covered (the quadrant shown was randomly chosen). **F**. Order of the frames temporally reversed (no sound in these cases).

Figure S6

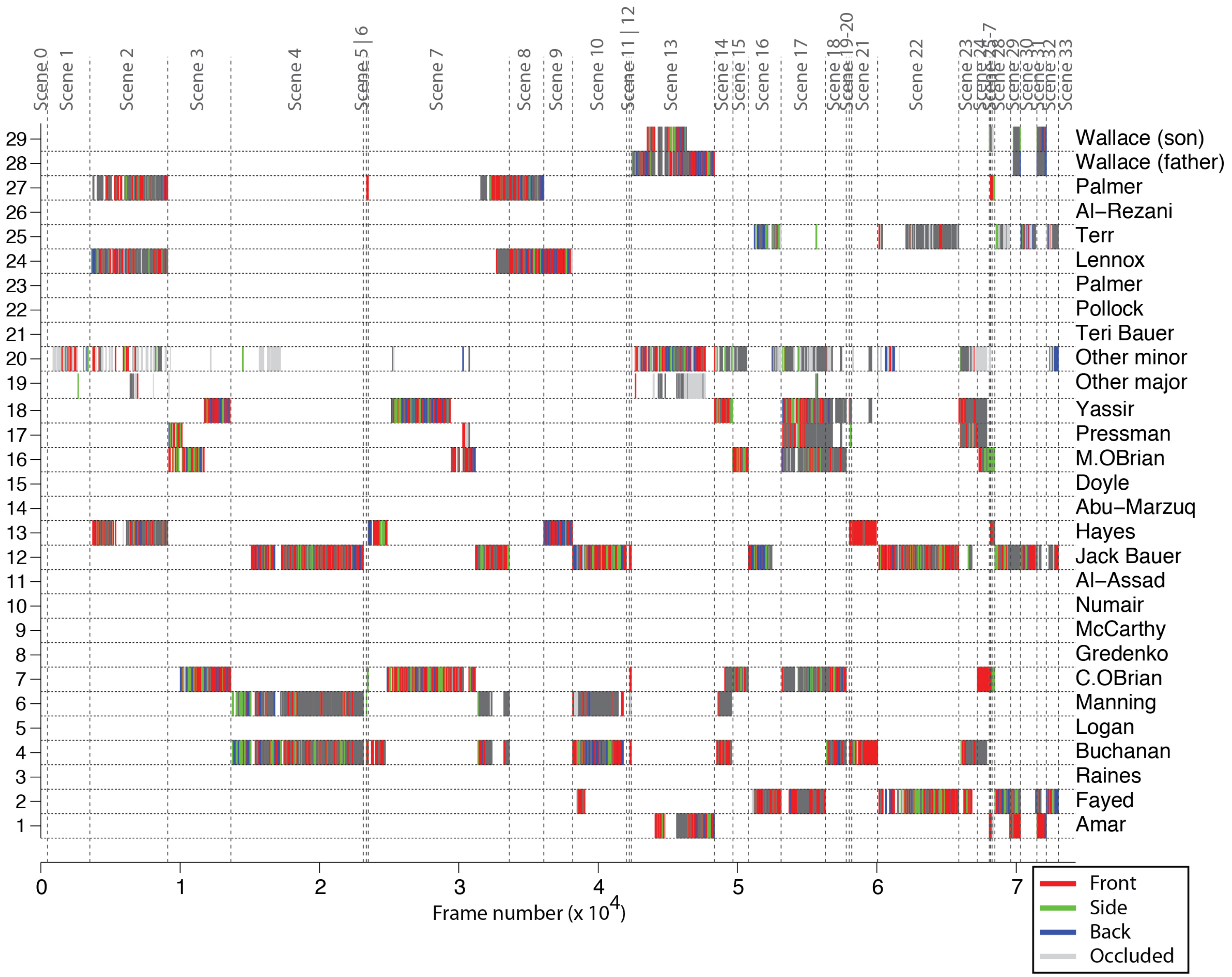

Figure S6. Presence of different characters during Episode 1 Annotations indicating the presence of 29 different characters during Episode 1. The x-axis indicates the position along the movie (in frames). Each row denotes a different character (names shown on the right). The color indicates the viewpoint (red=front, green=side, blue=back, gray=occluded). The vertical dashed lines indicate scene changes. Note that there are multiple shots (defined in **Figure S2**) within each scene. Similar annotations were used for Episode 2. Other aspects of the movie content were also annotated (**Methods**).

Figure S7

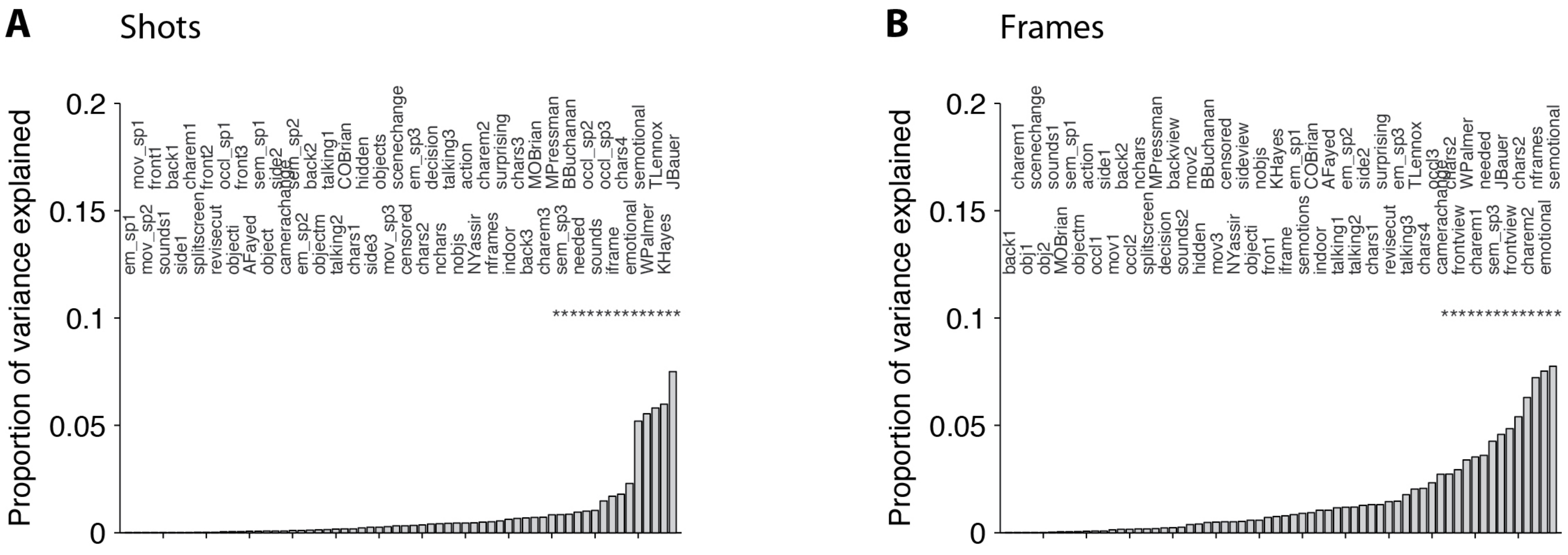

Figure S7. Contribution of different content variables to linear regression model of memorability
Expanding on the results shown in **Figure 5**, here we show the proportion of the variance explained by each content variable in the linear regression model to capture performance for shots (**A**) or frames (**B**). The content variables are described in **Tables S2-S3**. * denotes those content variables that showed a significant contribution.

Figure S8

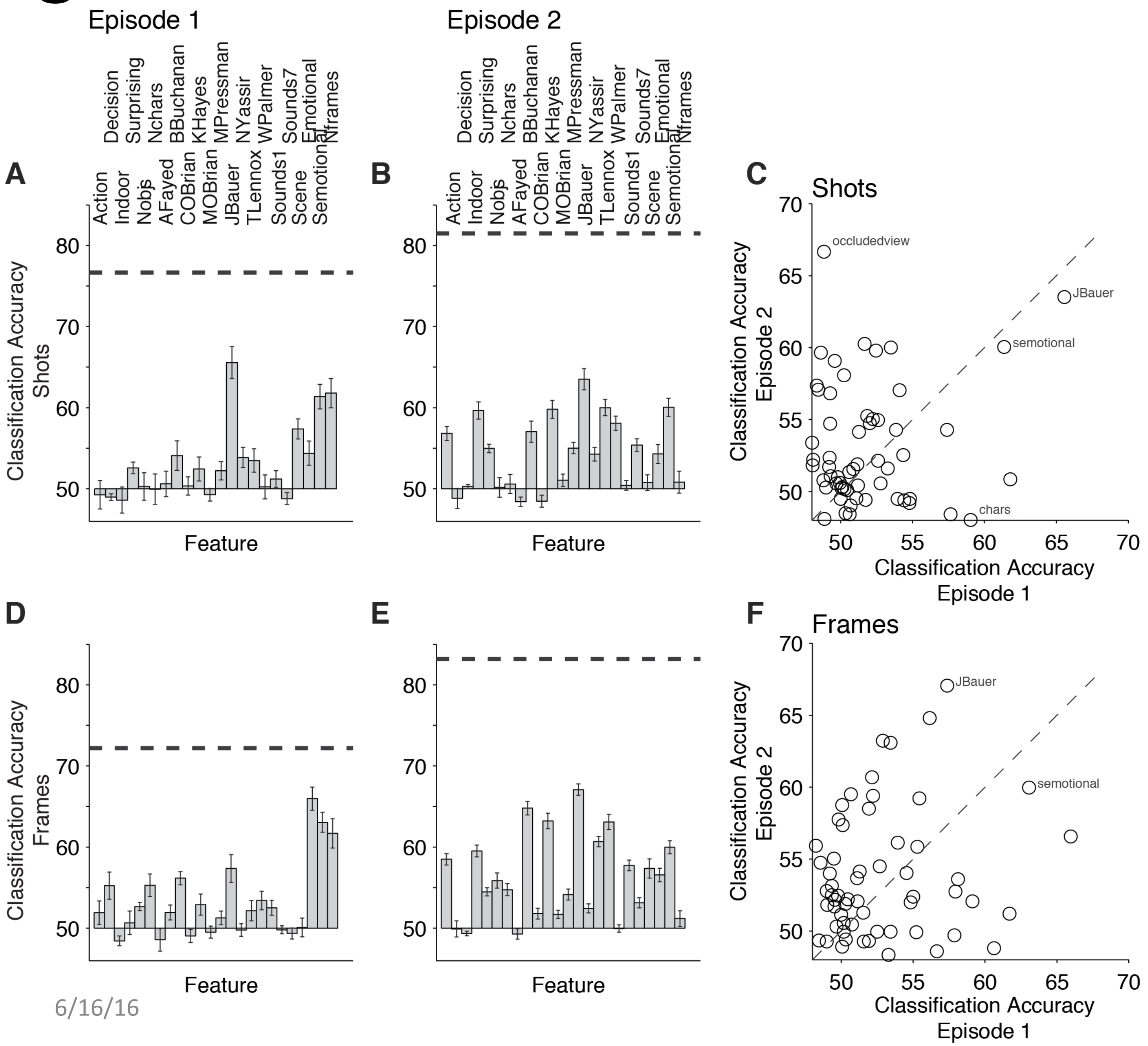

Figure S8. Machine learning decoding of memorability for each episode
A-B, D-E. Expanding on **Figure 7** in the main text, the classifier performance is shown here for each episode separately for movie shots (**A-B**) and single frames (**D-E**); format and conventions as in **Figure 7**. **C, F**. Classifier performance during episode 2 versus episode 1 using individual content properties for movie shots (**C**) or single frames (**F**).

Figure S9

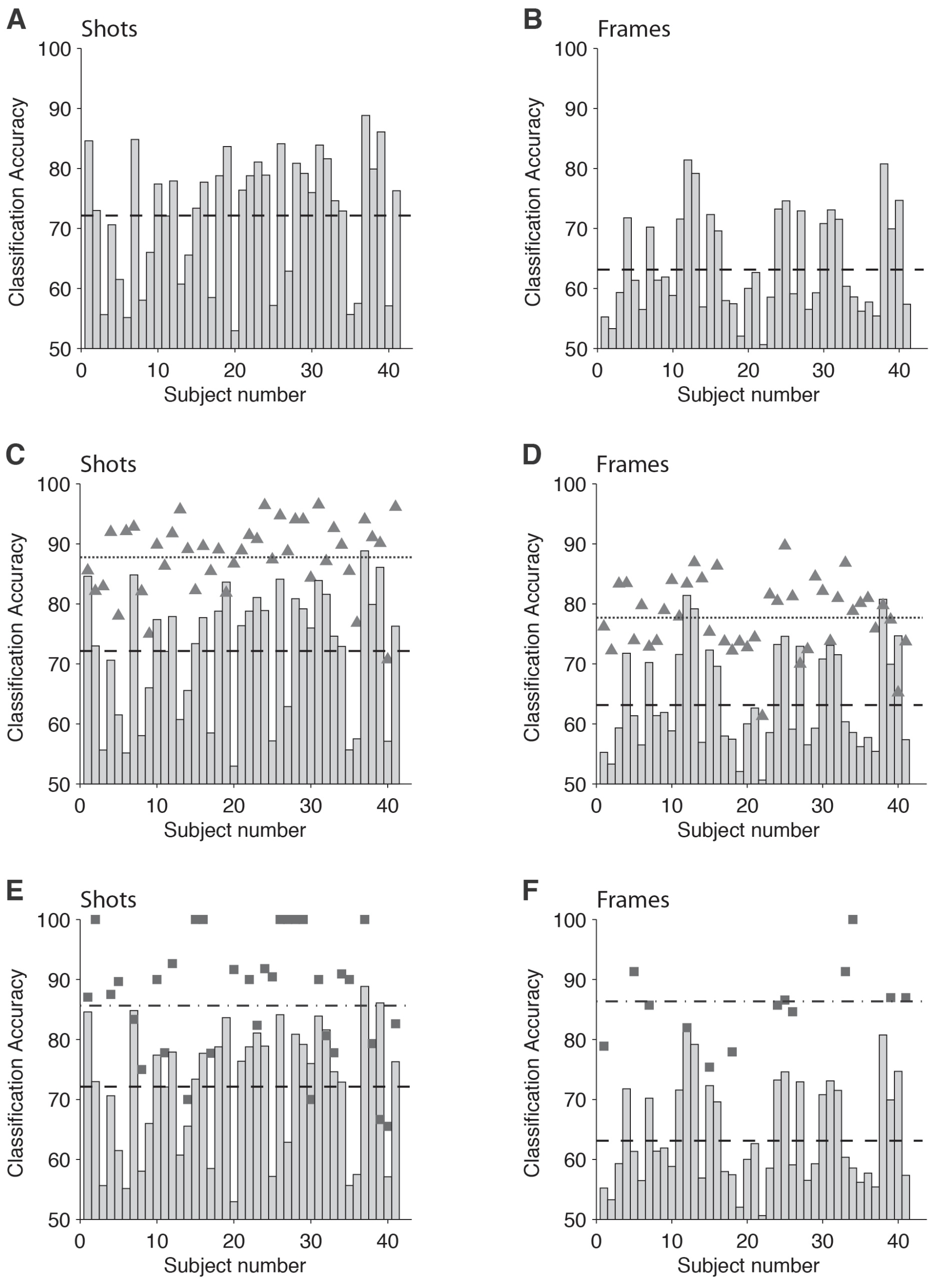

Figure S9. Machine learning decoding of memorability for individual subjects
Expanding on **Figure 7** in the main text, this plot shows the classifier performance for each individual subject for shots (**A**) and single frames (**B**). The horizontal dashed line indicates the mean across subjects. (**C-D**) We compared the machine learning approach against a classifier based on behavioral data where we used the mode across all *other* subjects to predict individual performance (“Mode”, triangles, averaged in the dotted line). (**E-F**) We compared the machine learning approach against a classifier based on behavioral data where we used repeat trials to predict individual performance for each subject (“Self”, squares, averaged in dashed-dotted line). The gray bars in **C-D** and **E-F** are exactly the same as in **A-B**.

Figure S10

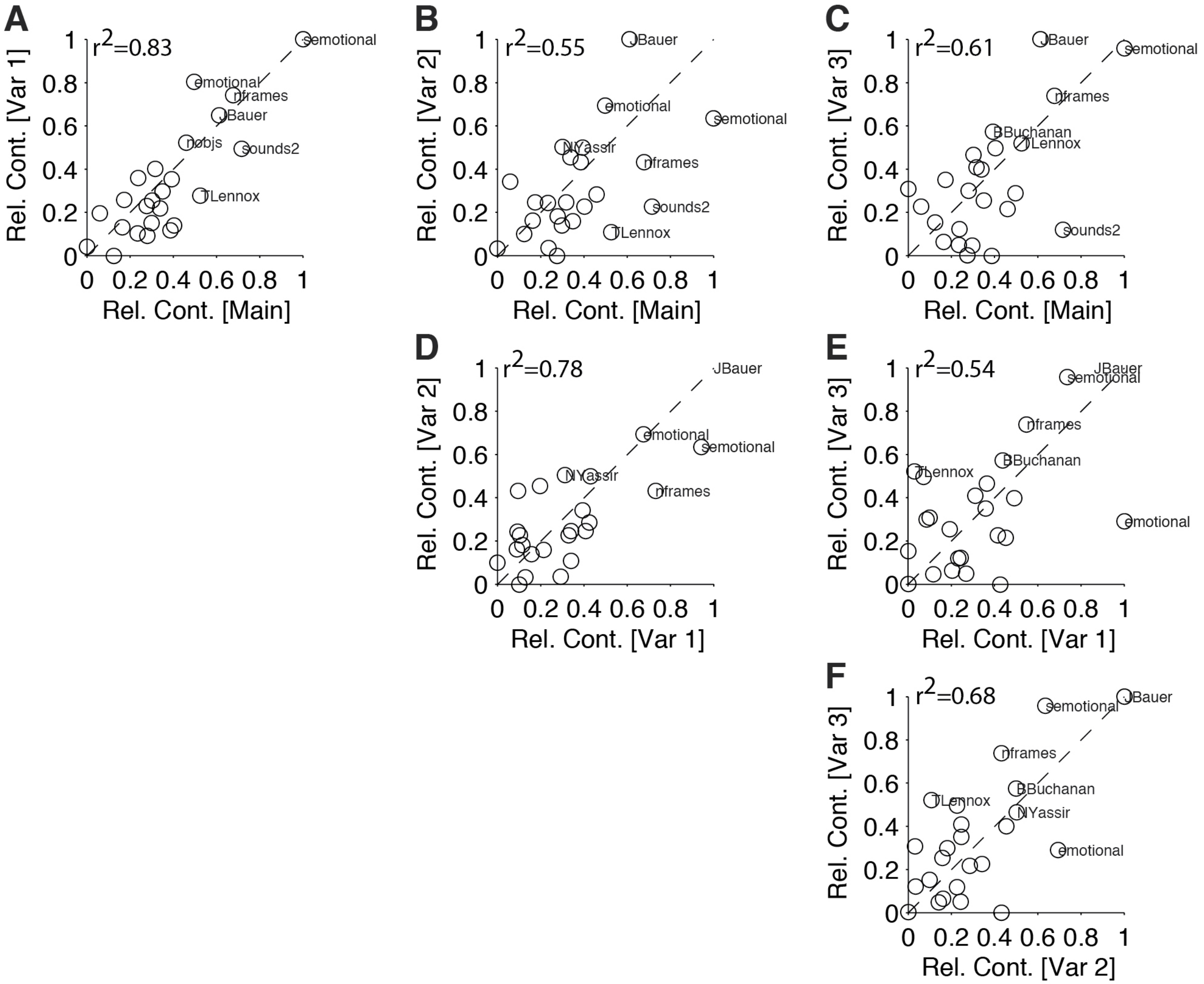

Figure S10. Comparison of relative contribution of content properties to classifier performance across experiments
The contribution of each content property was normalized to 0-1 for each experiment separately. Each scatter plot shows the relative contribution of each content property for two experiment variations. For clarity, the property name is only indicated for those contents that show a relative contribution > 0.5. The dashed line depicts the y=x diagonal for comparison purposes.
